# Endothelial Dysfunction in Youth-Onset Type 2 Diabetes: A Clinical Translational Study

**DOI:** 10.1161/CIRCRESAHA.124.324272

**Published:** 2024-07-29

**Authors:** Khaled Z. Abd-Elmoniem, Jehad H. Edwan, Katrina B. Dietsche, Alfredo Villalobos-Perez, Nour Shams, Jatin Matta, Leilah Baumgarten, Waleed N. Qaddumi, Sydney A. Dixon, Aruba Chowdhury, Michael Stagliano, Lilian Mabundo, Annemarie Wentzel, Colleen Hadigan, Ahmed M. Gharib, Stephanie T. Chung

**Affiliations:** National Institute of Diabetes and Digestive and Kidney Diseases, Biomedical Medical and Imaging Branch (K.Z.A., J.E., N.S., J.M., L.B., W.Q., A.M.G.), National Institutes of Health, Bethesda, MD.; Diabetes Endocrinology and Obesity Branch (K.B., A.V., S.D., A.C., M.S., L.M., S.T.C.), National Institutes of Health, Bethesda, MD.; Clinical Center (C.H.), National Institutes of Health, Bethesda, MD.; Hypertension in Africa Research Team (A.W.), North-West University, Potchefstroom.; South African Medical Research Council, Unit for Hypertension and Cardiovascular Disease (A.W.), North-West University, Potchefstroom.

**Keywords:** atherosclerosis, brachial artery, cardiovascular diseases, diabetes, type 2, pediatric obesity

## Abstract

**BACKGROUND:**

Youth-onset type 2 diabetes (Y-T2D) is associated with increased risk for coronary atherosclerotic disease, but the timing of the earliest pathological features and evidence of cardiac endothelial dysfunction have not been evaluated in this population. Endothelial function magnetic resonance imaging may detect early and direct endothelial dysfunction in the absence of classical risk factors (severe hyperglycemia, hypertension, and hyperlipidemia). Using endothelial function magnetic resonance imaging, we evaluated peripheral and coronary artery structure and endothelial function in young adults with Y-T2D diagnosed ≤5 years compared with age-matched healthy peers. We isolated and characterized plasma-derived small extracellular vesicles and evaluated their effects on inflammatory and signaling biomarkers in healthy human coronary artery endothelial cells to validate the imaging findings.

**METHODS:**

Right coronary wall thickness, coronary artery flow–mediated dilation, and brachial artery flow–mediated dilation were measured at baseline and during isometric handgrip exercise using a 3.0T magnetic resonance imaging. Human coronary artery endothelial cells were treated with Y-T2D plasma–derived small extracellular vesicles. Protein expression was measured by Western blot analysis, oxidative stress was measured using the redox-sensitive probe dihydroethidium, and nitric oxide levels were measured by 4-amino-5-methylamino-2’,7’-difluororescein diacetate.

**RESULTS:**

Y-T2D (n=20) had higher hemoglobin A1c and high-sensitivity C-reactive protein, but similar total and LDL (low-density lipoprotein)-cholesterol compared with healthy peers (n=16). Y-T2D had greater coronary wall thickness (1.33±0.13 versus 1.22±0.13 mm; *P*=0.04) and impaired endothelial function: lower coronary artery flow–mediated dilation (−3.1±15.5 versus 15.9±17.3%; *P*<0.01) and brachial artery flow–mediated dilation (6.7±14.7 versus 26.4±15.2%; *P*=0.001). Y-T2D plasma–derived small extracellular vesicles reduced phosphorylated endothelial nitric oxide synthase expression and nitric oxide levels, increased reactive oxygen species production, and elevated ICAM (intercellular adhesion molecule)–mediated inflammatory pathways in human coronary artery endothelial cells.

**CONCLUSIONS:**

Coronary and brachial endothelial dysfunction was evident in Y-T2D who were within 5 years of diagnosis and did not have severe hyperglycemia or dyslipidemia. Plasma-derived small extracellular vesicles induced markers of endothelial dysfunction, which corroborated accelerated subclinical coronary atherosclerosis as an early feature in Y-T2D.

**REGISTRATION:**

URL: https://www.clinicaltrials.gov; Unique identifier: NCT02830308 and NCT01399385.

**Novelty and Significance**

**What Is Known?**

- Youth-onset type 2 diabetes is an aggressive chronic condition that is rapidly increasing worldwide. Within 2 years, more than 1 in 2 youths with type 2 diabetes require intensive glucose-lowering therapy and have a high risk for early development of diabetes-related complications.
- The timing and early features of coronary heart disease development are not yet known. To help prevent heart disease in youth with type 2 diabetes, more information is needed on when and how heart disease develops so that targeted prevention strategies can be developed to mitigate the high risk.

**What New Information Does This Article Contribute?**

- Young adults with youth-onset type 2 diabetes had early signs of coronary heart disease and vasoconstriction to handgrip exercise. These findings were confirmed by laboratory experiments using blood samples containing small extracellular vesicles that increased the markers of endothelial dysfunction in normal human coronary endothelial cells.
- These findings of impaired endothelial function are important because they occurred in adequately treated young adults with type 2 diabetes for 5 years.
- This was the first study to show impairment in the structure and function of heart and arm vessels using both magnetic resonance imaging and blood tests of small extracellular vesicles in youth-onset type 2 diabetes.
- This type of bench-to-bedside research, using noninvasive magnetic resonance imaging and ex vivo translational analyses, is ideal for identifying the early features of heart disease in young people without using cardiac catheterization.

The number of youth with obesity and type 2 diabetes is rapidly increasing worldwide, and these youths have a greater chance of developing diabetes-related problems. Young adults with type 2 diabetes may develop early heart disease, but there is insufficient data on when or how heart disease develops in at-risk youth. In this study, magnetic resonance imaging was used to examine the size and function of 2 major blood vessels: the right coronary artery and the right brachial artery. We found that young adults with type 2 diabetes had thicker right coronary arteries and abnormally restricted blood flow in the heart and arm during a simple exercise test. In laboratory experiments, we confirmed our findings by showing that blood samples from young adults with diabetes contained small extracellular vesicles that could impair vessel function. This study is important because early signs of heart disease were observed in young adults who were on treatment and had diabetes for ≤5 years. This bench-to-bedside study helps to confirm early features of heart disease in youth-onset type 2 diabetes and supports future studies to prevent the progression of heart disease in at-risk youth.

**In This Issue, see p 635**

**Meet the First Author, see p 636**

Youth-onset type 2 diabetes (Y-T2D) is a rapidly progressive disease associated with severe insulin resistance and β-cell dysfunction, which distinctly differs from adult-onset type 2 diabetes (A-T2D) and is associated with the early development of cardiometabolic complications.^1–3^ Globally, the rising prevalence rates of Y-T2D parallel the pediatric obesity epidemic, placing an undue burden on underserved and minoritized racial and ethnic groups.^4–7^ Early epidemiological reports indicate that coronary atherosclerotic disease (CAD)—the leading cause of death in A-T2D—may afflict young adults with Y-T2D during their peak productivity years, despite a short disease duration. However, the tempo of CAD and coronary endothelial dysfunction—recognized as the initial stage in CAD development—in Y-T2D is unknown. Identifying these early features of CAD and coronary endothelial dysfunction directly and noninvasively in Y-T2D is critical and timely because it could serve as a practical early indicator of CAD to support early screening and CAD prevention programs.

Endothelial function may be measured in the peripheral or central vasculature. Peripheral endothelial function can be assessed noninvasively via a vasodilatory response to external noxious stimuli in the brachial artery or finger capillaries. Centrally, endothelial function is assessed using invasive coronary artery catheterization or noninvasively with cardiac magnetic resonance spectroscopy.^8^ In A-T2D, central vascular measurement of coronary endothelial dysfunction is an established cardiac risk marker; early CAD has been characterized by both impaired coronary vasodilation in response to nitric oxide (NO) stimuli and increased coronary wall thickness (CWT) in A-T2D.^9^ In youth-onset type 1 diabetes, endothelial dysfunction occurred after multiple years of hyperglycemia and advanced disease.^10,11^ In contrast to type 1 diabetes, up to 25% of Y-T2D have traditional cardiovascular risk factors at diagnosis, and severe insulin resistance could be associated with endothelial dysfunction at an early disease stage. However, coronary artery data are limited in Y-T2D because coronary catheterization is invasive and not justifiable in this young population without end-stage coronary artery disease. The few studies assessing endothelial function in Y-T2D were limited to evaluating peripheral vasculature only, and none have directly interrogated the coronary arteries in this population. Some, but not all studies,^12^ showed that compared with age-matched and body mass index (BMI)–matched peers, Y-T2D had modestly impaired peripheral endothelial function that progressively worsened with time.^13,14^ However, the interrelation between peripheral assessments of endothelial function and the early atherosclerotic shift in smaller coronary arteries remains unclear.^15,16^

Therefore, this study adopted a noninvasive, yet powerful, approach to directly visualize coronary structure and endothelial function while validating the cardiac magnetic resonance imaging (MRI) findings with biological experiments to investigate the natural history of CAD in Y-T2D. Ex vivo analysis of small extracellular vesicle (ECV) cargo derived from Y-T2D was utilized to identify molecular changes in cultured human coronary artery endothelial cells (HCAECs). Small ECV–mediated mechanisms of endothelial dysfunction have been demonstrated in previous research in adults; however, no data are available in youth. Small ECVs containing diacyl-glycerol directly suppressed eNOS (endothelial NO synthase) activity, inducing an inflammatory cascade through intercellular crosstalk.^17,18^ In A-T2D, small ECVs were implicated in mediating endothelial function via proinflammatory cytokine production and oxidative stress.^19,20^

This translational pilot study was designed to identify, quantify, and evaluate CAD and endothelial function in Y-T2D using clinical noninvasive MRI examination of coronary and peripheral vessels, coupled with ex vivo validation analysis of the effect of Y-T2D plasma–derived small ECVs on coronary endothelial cells. We hypothesized that young adults with Y-T2D, compared with lean healthy age-matched peers, would exhibit greater CWT and impaired peripheral and coronary arterial endothelial function identified by noninvasive MRI conducted at rest and after isometric handgrip exercise. To validate the imaging findings, we isolated Y-T2D plasma–derived small ECVs from patients with Y-T2D to assess their potential to induce endothelial dysfunction in HCAECs from healthy young adult donors. We further hypothesized that these Y-T2D plasma–derived small ECVs would alter cellular function by reducing eNOS signaling, impairing NO production, and promoting the expression of inflammatory cytokines.

## METHODS

### Data Availability

The data that support the findings of this study are available from the corresponding author upon reasonable request.

#### Participants and Cardiac MRI Study

Young adults with Y-T2D and lean healthy young adult volunteers were prospectively recruited for cross-sectional cardiovascular imaging and assessment of CAD at the National Institutes of Health Clinical Center (https://www.clinicaltrials.gov; unique identifiers: NCT02830308 and NCT01399385). Y-T2D was ≥18 years of age with a ≤5-year diagnosis of Y-T2D or prediabetes defined by the American Diabetes Association criteria.^21^ Participants were excluded if they had known congenital or acquired heart disease, positive autoantibodies to glutamic acid decarboxylase-65 or insulinoma-associated protein-2, or a contraindication to MRI. There were no restrictions on medication use or the presence of diabetes-related comorbidities for Y-T2D. Lean young adults were age-matched (±3 years) and self-identified as healthy without chronic diseases or medication use. Written informed consent was obtained before participation, the protocols were approved by the Institutional Review Board of the National Institute of Diabetes and Digestive and Kidney Diseases, and the study was conducted in compliance with the Declaration of Helsinki.

Participants were evaluated after a 10- to 12-hour overnight fast, complete history and physical examinations, anthropometrics, blood pressure measurements, and fasting blood sample collection for glucose, insulin, lipid panel, hs-CRP (high-sensitivity C-reactive protein), and small ECV analysis. Fasting glucose, lipid panel, and insulin concentrations were measured in serum using the Roche Cobas 6000 analyzer (Roche Diagnostics, Indianapolis, IN). Hemoglobin A1c (HbA1c) was measured using the HPLC D10 instrument (Bio-Rad) and hs-CRP by immunoturbidometric method assayed (Abbott Architect).

Early CAD was assessed by CWT obtained from MRI of the proximal right coronary artery during diastole as previously described.^22^ In summary, exams were performed using a commercial 3.0T whole-body MR scanner (Ingenia Elition, Philips, Best, the Netherlands) with a 32-element surface coil for signal reception. Double-oblique scout scanning was performed to identify relevant portions of the native coronary artery and to ensure that slice orientation was perpendicular to the coronary vessel.

Endothelial function was assessed by MRI; brachial artery flow–mediated dilation (BraFMD), and coronary artery flow–mediated dilation were measured in response to an NO-mediated exercise utilizing an isometric hand grip exercise while in the MR scanner.^23,24^ Anatomic cross-sectional bright blood images of these vessels were obtained at baseline and during 5 minutes of continuous isometric handgrip exercise at a constant 30% of each subject’s maximum grip strength. An MRI-compatible in-house developed handgrip system based on BIOPAC MP150 hardware, and acknowledged data acquisition software (Goleta, CA) was used. The system has an audiovisual continuous monitoring feedback mechanism to inform the participant and the MRI operator during the 5-minute workout exercise about the participant’s adherence to gripping instructions and enforcement of the required gripping strength.^24^ This method has previously demonstrated an intraobserver, interobserver, and interexamination agreement for coronary vessel wall thickness measurements of 0.98, 0.97, and 0.92, respectively. Observers were blinded to the participant’s status at the time of MRI image analysis. Baseline and postisometric hand grip exercise images of coronary cross-sectional area were calculated using region-growing and full-width at half-maximum criterion and a semiautomated in-house custom-built software tool developed using MATLAB, version 10 (Mathworks, Natick, MA) with similar functionalities to previously published tools.^25^ Segments with poor image quality (blurring due to artifact/subject motion) on either the baseline or postisometric hand grip exercise exams were excluded from the analysis.

#### Blood Collection, Plasma, and Small ECV Isolation

To characterize and validate the presence of isolated ECVs, we followed the guidelines of the International Society for Extracellular Vesicles.^26^ Blood from 10 consecutive patients with Y-T2D and 1 patient with A-T2D was collected using a syringe containing 50 µL of 0.5-M EDTA (ethylenediaminetetraacetic acid) into BD Vacutainer Venous Blood Collection Tubes (cat. No. 367525, BD, Franklin Lakes, NJ), which also contained EDTA. For plasma separation, the blood was maintained at room temperature (25 °C) for 15 minutes and then centrifuged at 1000*g* at 20 °C for 10 minutes. The supernatant comprising the upper plasma phase was carefully transferred to a new tube. This supernatant was recentrifuged at 3000*g* at 20 °C for 15 minutes and filtered through a 0.45-µm filter. Small ECVs were isolated from the filtrate using a total small ECV isolation kit (Invitrogen from plasma) according to the manufacturer’s instructions. The kit was used in conjunction with proteinase K treatment to selectively degrade potential contaminating particles and proteins.

For comparison, we isolated small ECVs from plasma obtained from 3 healthy young adult donors from Lonza (Frederick, MD) to serve as negative controls, and these samples were used separately, not pooled, when treating HCAEs. Plasma-derived small ECV processing was identical for all samples and completed by a blinded investigator (J.E.).

The presence of small ECVs was confirmed based on their morphology and size using transmission electron microscopy (JEM 1200EX [JEOL]). Small ECV identification was confirmed using antibodies specific to common protein markers (CD9 and CD63) found on exosomal surface membranes (a subtype of small ECVs). A nonspecific IgG isotype antibody was used as a negative control. All EM images were analyzed on ImageJ, version 1.49 (National Institutes of Health, Bethesda, MD).

#### Western Immunoblotting Analysis of Small ECV–Specific Markers

To further verify the purity of small ECV isolation, immunoblotting analysis was performed on small ECVs from healthy donors, and Y-T2D cell lysate from HCAEC was used as a negative control for purity analysis. The antibodies used for immunoblotting included small ECV–specific anti-TSG101 (tumor susceptibility 101), anti-CD9, anti-CD63, anti-CD81, and Alix (ALG-2-interacting protein X). Additionally, anti-ApoE (apolipoprotein E) was used to confirm the absence of lipoproteins that might have been included in the extraction process, and anti-calnexin, a nonsmall ECV endoplasmic reticulum–related antibody, was used according to guidelines.^26^ This combination of techniques ensures the specificity and purity of the isolated small ECVs.

#### Tracing Small ECVs Uptake by HCAECs

We utilized a visualization technique using SYTO RNASelect cell stain to label small ECVs containing RNA, tracking their uptake by HCAECs after 48 hours of incubation.^26^ This stain, which emits green fluorescence, selectively binds to RNA within small ECVs, enabling the examination of their colocalization within the HCAECs. Two control groups were used in this experiment: (1) HCAECs without small ECVs or dye and (2) cells treated with dye only. Additionally, the HCAECs were stained with Phalloidin (Thermo Fisher Scientific), which emits red fluorescence to highlight cell structure, and 4′,6-diamidino-2-phenylindole, which emits blue fluorescence to mark cell nuclei, facilitating their visualization under fluorescent microscopy.

#### Effects of Isolated Small ECVs on HCAEC

To investigate the effects of isolated small ECVs on endothelial function, we quantified the protein expression of eNOS, a key enzyme regulating NO production in endothelial cells, and ICAM-1 (intercellular adhesion molecule-1), an immunoglobin critical for leukocyte adhesion and migration. HCAECs were treated with equal concentrations of small ECVs derived from both healthy donors and individuals with Y-T2D. As controls, we used a small ECV-depleted fraction (the supernatant remaining after small ECV isolation from Y-T2D plasma) and HCAECs without any small ECV treatment. In a subsequent experiment, HCAECs treated with Y-T2D-derived small ECVs and those without small ECV treatment (as control) were analyzed to measure total NO and reactive oxygen species (ROS) levels using flow cytometry. An imbalance between NO and ROS is a recognized indicator of endothelial dysfunction. The interaction between ROS and NO, where ROS can quench and inactivate NO, highlights the complex relationship between vascular oxidative stress, endothelial dysfunction, and the increased risk of cardiovascular pathologies.^27^

#### Statistical Analysis

The clinical study was designed to evaluate CWT in Y-T2D compared with lean peers. Sample size estimates (n=14 per group) were based on estimates from our prior publication^28^ and were designed to detect CAD as a mean CWT difference of 0.31 mm and SD of difference of 0.2 mm (mean±SD) with between-group differences, β≥0.9 and α=0.05. Data are presented as mean±SD, except where otherwise indicated. Continuous variables were compared using the Student *t* test or rank-sum analysis and categorical variables using the Fischer exact tests. Normality was determined by visual inspection of histograms. Triglyceride, insulin, and C-reactive protein concentrations were natural logarithmically transformed before analysis. Spearman correlations (r) were used to determine the associations between cardiometabolic variables and markers of endothelial function. For each experimental condition, images were chosen based on their ability to represent typical results observed across multiple independent experiments. No statistical corrections for multiple analyses were performed because this was an exploratory analysis. Western blots were quantified using the ImageJ program. Statistical significance of individual plasma–derived small ECVs was compared with controls (HCAECs without small ECV treatment) using the Mann Whitney *U* tests. Statistical analyses for clinical variables were performed using STATA (version 16.1; Stata Corp, College Station, TX), while analyses related to small ECVs data were conducted using GraphPad, Prism V9.0 (GraphPad Software, San Diego, CA). Statistical significance was established at *P*<0.05.

## RESULTS

Twenty-one Y-T2Ds were screened, 20 enrolled, and 15 completed the cardiac MRI. Five Y-T2Ds attempted the cardiac MRI but were unable to complete testing because of discomfort during the scan. Sixteen healthy lean young adults with cardiac MRI data were age-matched (±3 years) to the 15 Y-T2D. Figure S1 illustrates the participant flow diagram.

### Participant Characteristics

The participant demographic and cardiometabolic characteristics are presented in the Table. Y-T2D had higher HbA1c, BMI, triglyceride concentrations, blood pressure, heart rate, and hs-CRP compared with lean age-matched healthy young adults. LDL (low-density lipoprotein) cholesterol concentrations were comparable between groups. All Y-T2Ds were prescribed at least 1 glucose-lowering medication, 20% were prescribed antihypertensive therapy, and no participants were taking cholesterol-lowering medications. Demographic and metabolic characteristics of participants who completed MRI (Table S1) and small ECV analysis (n=10; Table S2) were similar to the entire Y-T2D group.

**Table.**
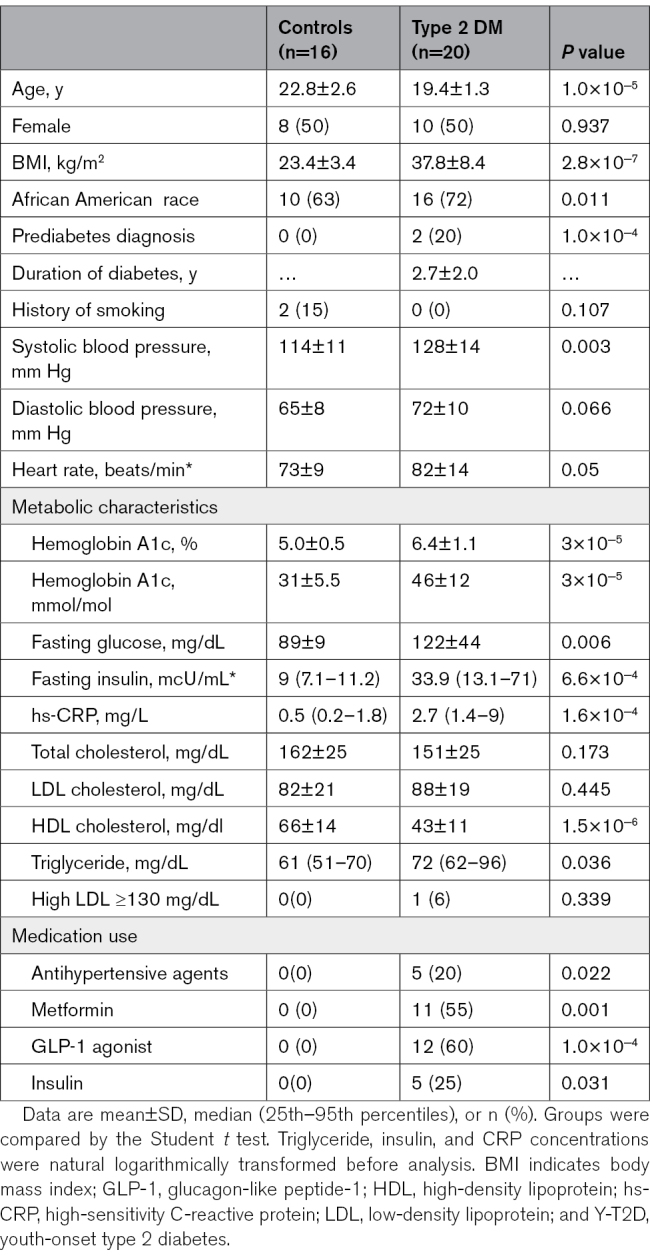
Demographic and Cardiometabolic Characteristics in Y-T2D and Healthy Lean Young Adults

#### Impaired Coronary and Peripheral Arterial Endothelial Function in Y-T2D

**Figure 1.**
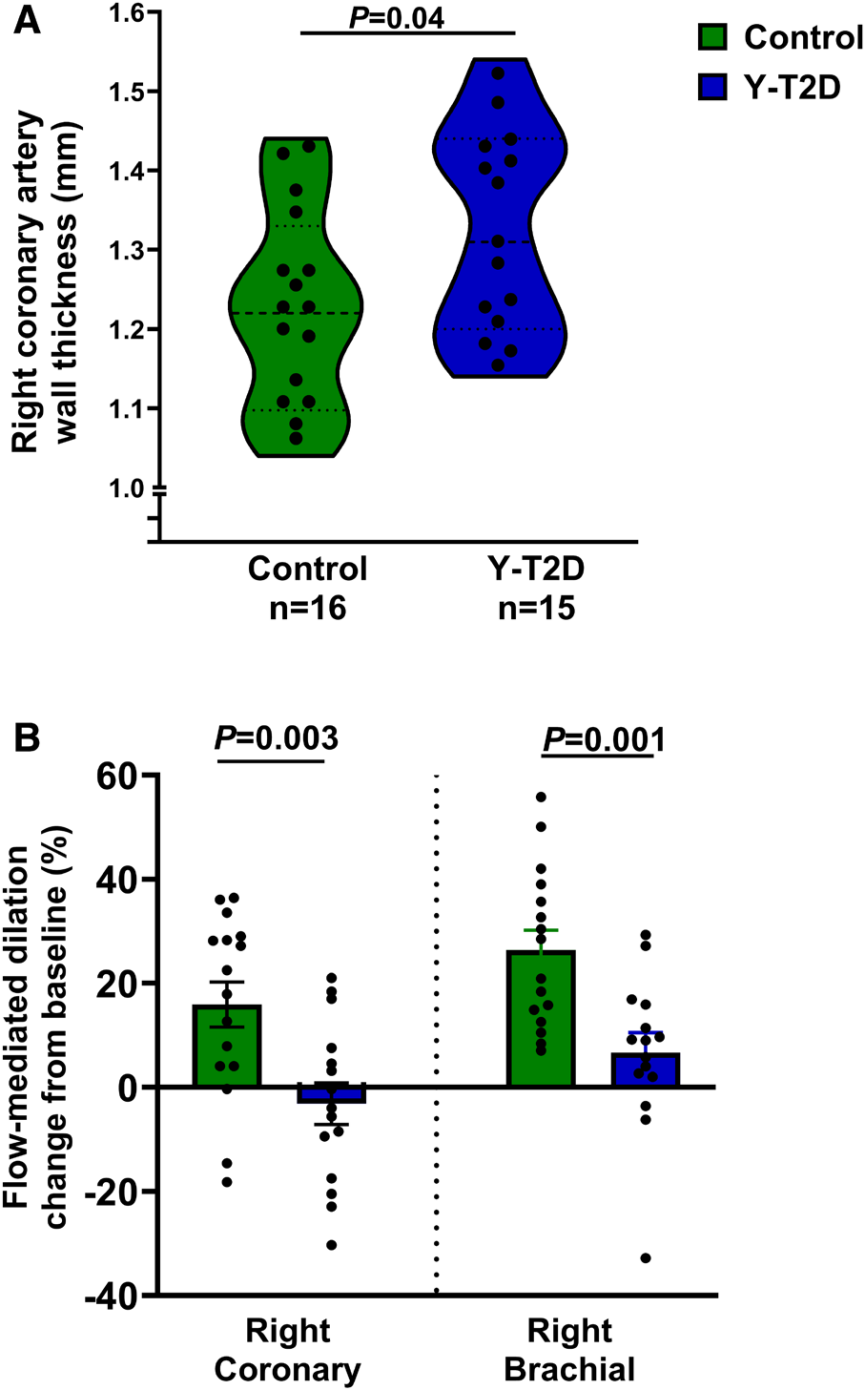
**Coronary arterial wall structure and function in youth-onset type 2 diabetes (Y-T2D) and lean healthy age-matched peers (control).** Right coronary wall thickness (**A**) and lumen FMD percent change (**B**) from baseline in the right coronary and brachial arteries during the isometric handgrip exercise in control (green) compared with Y-T2D (blue). Data are presented as violin plots (**A**) and bar graphs mean (**B**) with individual data points (black circles). Groups were compared with the Student *t* test. *P*<0.05 was considered significant and shown inline.

The Y-T2D had 10% greater right CWT compared with lean healthy controls (Figure 1A). Additionally, there was an impairment of arterial endothelial vasodilation in response to isometric handgrip exercise. BraFMD showed an 80% reduction, and the coronary artery flow–mediated dilation demonstrated vasoconstriction (Figure 1B) in Y-T2D compared with healthy controls. Coronary artery flow–mediated dilation correlated with BrFMD (r=0.5; *P*=0.005). Table S3 shows CWT and endothelial function associations with cardiometabolic markers. HbA1c was associated with greater CWT (r=0.4; *P*=0.04) and arterial endothelial dysfunction (lower coronary artery flow–mediated dilation and BraFMD; both r=−0.5; *P*<0.01). Brachial artery vasodilation was also associated with BMI (r=0.4; *P*=0.04) and tended to associate with blood pressure (r=0.4; *P*=0.07).

#### Y-T2D Small ECVs Induced Markers of Endothelial Dysfunction in HCAECs

**Figure 2.**
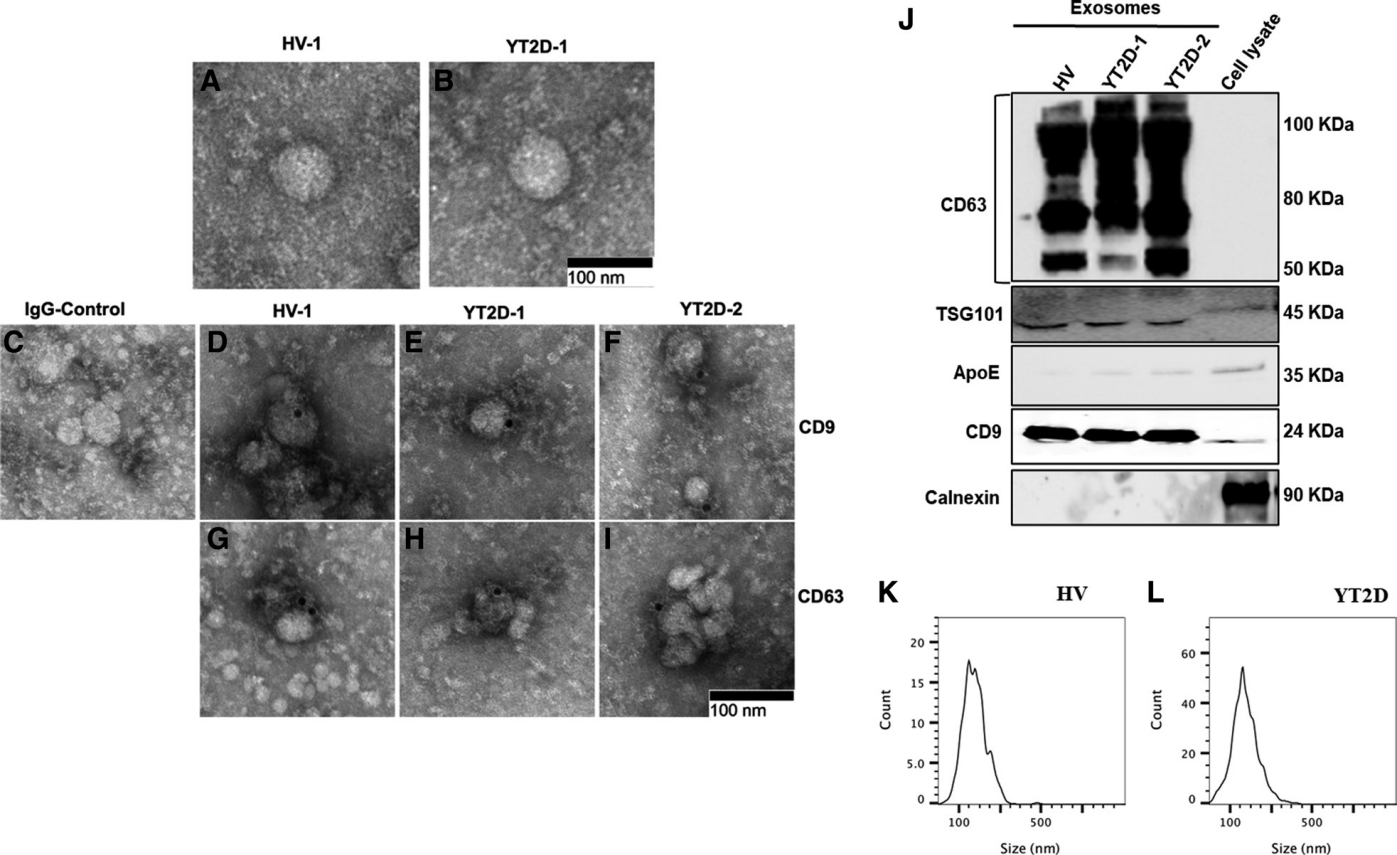
**Characterization of plasma-derived small extracellular vesicles.** Plasma-derived small extracellular vesicles (small ECVs) were isolated from healthy donor volunteers (HVs; n=1) and youth-onset type 2 diabetes (Y-T2D; n=2), then stained with uranyl acetate (**A** and **B**), labeled with IgG control, illustrating the baseline distribution of particles without specific targeting (**C**), or 10-nm immunogold particles using antibodies against the exosomal membrane markers, CD9 (**D**–**F**) and CD63 (**G**–**I**), and stained with uranyl acetate. Small ECVs were imaged by electron microscopy. Scale bar, 100 nm. **J**, Western blot analysis was conducted on lysates sourced from the small ECVs fraction and cell pellet, utilizing antibodies against CD63, TSG101 (tumor susceptibility 101), ApoE (apolipoprotein E), CD9, and calnexin. The expressions of TSG101, CD9, and CD63 were predominantly identified in plasma-derived small ECV extracts; calnexin was not detected, confirming the exclusion of endoplasmic reticulum components. ApoE and calnexin were primarily observed in cell lysates and traces of observed in small ECV extracts. **K** and **L**, Nanoparticle tracking analysis quantification of small ECV size. The *x* axis represents the small ECV size in nanometers (nm). The *y* axis represents the frequency (count) of particles found at each size interval.

**Figure 3.**
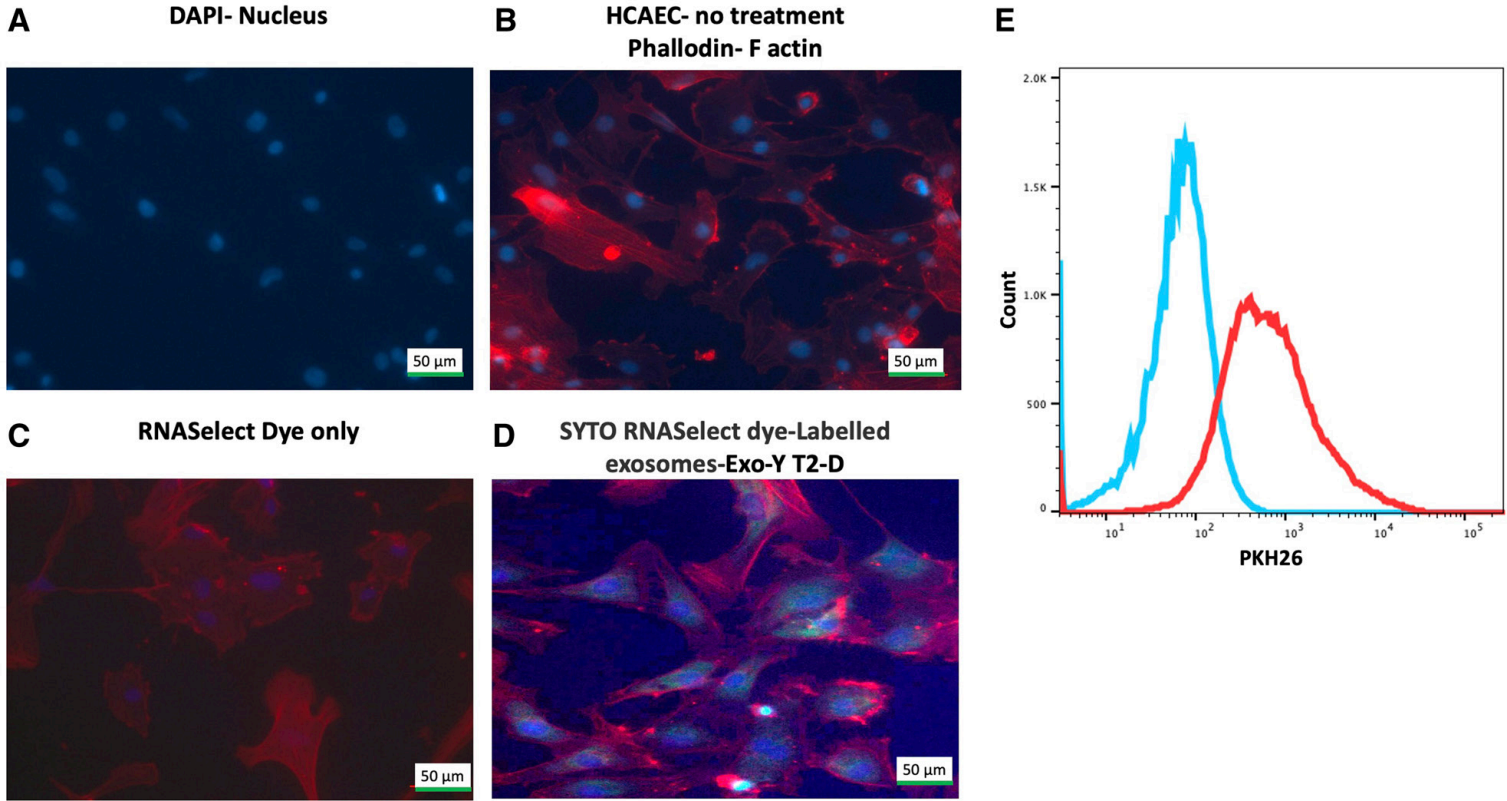
**Visualization of the uptake of small extracellular vesicles by human coronary artery endothelial cells (HCAECs). A**, HCAECs were stained with 4′,6-diamidino-2-phenylindole (DAPI, blue) to visualize nuclei and (**B**) phallodin (red) for actin filaments. **C**, HCAECs were incubated with RNASelect dye only. **D**, HCAECs were incubated with SYTO RNASelect dye-labeled small extracellular vesicles (green) and imaged at 48 h. Scale bar, 50 µm. **E**, Flow cytogram showing the shift in fluorescence intensity in HCAECs after incubation with PKH26-labeled plasma–derived youth-onset type 2 diabetes (Y-T2D) small extracellular vesicles (red) and control (blue).

Plasma-derived small ECVs from both patients with Y-T2D and healthy donor controls were successfully isolated, exhibiting round, cup-shaped, or saucer-like structures (Figure 2A and 2B). Immunogold staining confirmed the presence of small ECV–specific markers, CD9 and CD63, thus verifying the identity of these particles as small ECVs in samples from both groups (Figure 2D through 2I). Nonspecific IgG antibodies showed no binding to the small ECVs, indicating that the specificity of the CD9 and CD63 antibodies was unique to small ECV identification. Western blot analysis further validated the presence of TSG101, CD9, CD63, CD81, and Alix markers in both healthy donor volunteers and Y-T2D small ECVs (Figure 2J; Figure S2). Notably, calnexin was absent, confirming the exclusion of endoplasmic reticulum components. However, despite our efforts to minimize contamination, traces of ApoE were still detectable in our assays (Figure 2J). This observation suggests that while our small ECV isolation method significantly reduced the presence of major lipoproteins, it did not eliminate them, underscoring the need for cautious interpretation of the purity assessments. Nanoparticle tracking analysis revealed a size range of 50 to 180 nm for the small ECVs, consistent with guidelines (Figure 2K and 2L). Further analysis demonstrated the uptake of labeled small ECVs by HCAECs after 48 hours of incubation (Figure 3A and 3D). This uptake was visualized using RNASytoSelect dye and PKH26 lipid dye fluorescent imaging, showing that HCAECs incorporated green fluorescent labeled small ECVs (Figure 3D), but no green fluorescence was detected in cells without small ECV treatment (Figure 3C). Additionally, the interaction of Y-T2D-derived small ECVs with target HCAECs was quantified using the PKH26 lipid probe, a fluorescent lipid marker commonly used in flow cytometry to track small ECV uptake.^29^ Approximately 86% of HCAECs displayed uptake of fluorescent small ECVs within 24 hours (Figure 3E), indicating efficient cell membrane crossing into HCAEC and specific labeling by the small ECVs.

**Figure 4.**
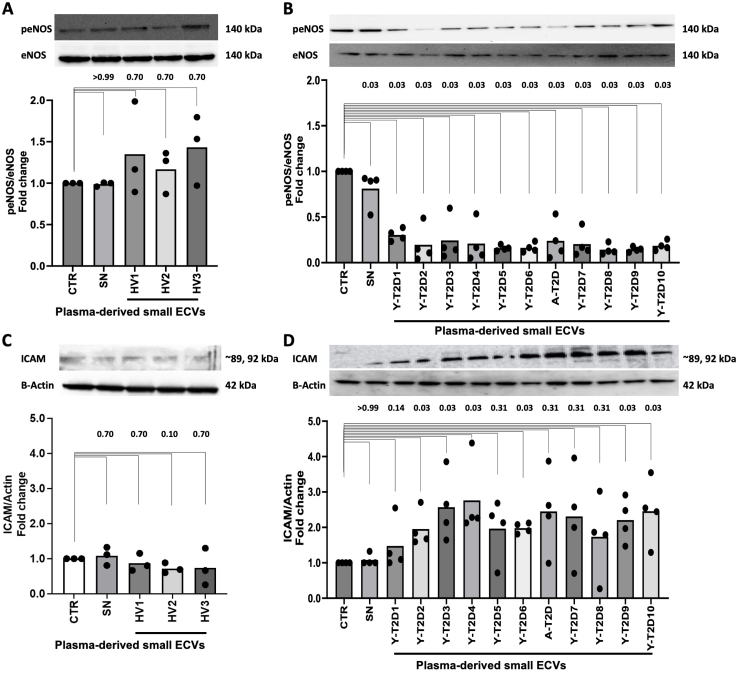
**Protein expression analysis in human coronary artery endothelial cells (HCAECs) after small extracellular vesicles (small ECVs) treatment.** Small ECVs were isolated from the plasma of healthy donor volunteers (HVs; n=3), youth-onset type 2 diabetes (Y-T2D; n=10), and adult-onset type 2 diabetes (A-T2D; n=1) and subsequently used to treat HCAECs over a period of 48 h. After treatment, the expression of target proteins, peNOS (phosphorylated endothelial nitric oxide synthase) and ICAM (intercellular adhesion molecule), was assessed. **A** and **B**, Western blot analysis showing the fold change in peNOS relative to total eNOS (endothelial nitric oxide synthase) after treatment with plasma-derived small ECVs. **C** and **D**, Western blot analysis depicting the fold change in ICAM-1 relative to β-actin after small ECV treatment. Individual data (black circles) are presented with bar graphs for mean, with 3 replicates for **A** and **C** and 4 replicates for **B** and **D**. Mann Whitney *U* tests were used to compare vs control (CTR, untreated cells) and supernatant (SN; HCAECs treated with SN-depleted small ECVs). *P*<0.05 was considered significant and shown inline.

**Figure 5.**
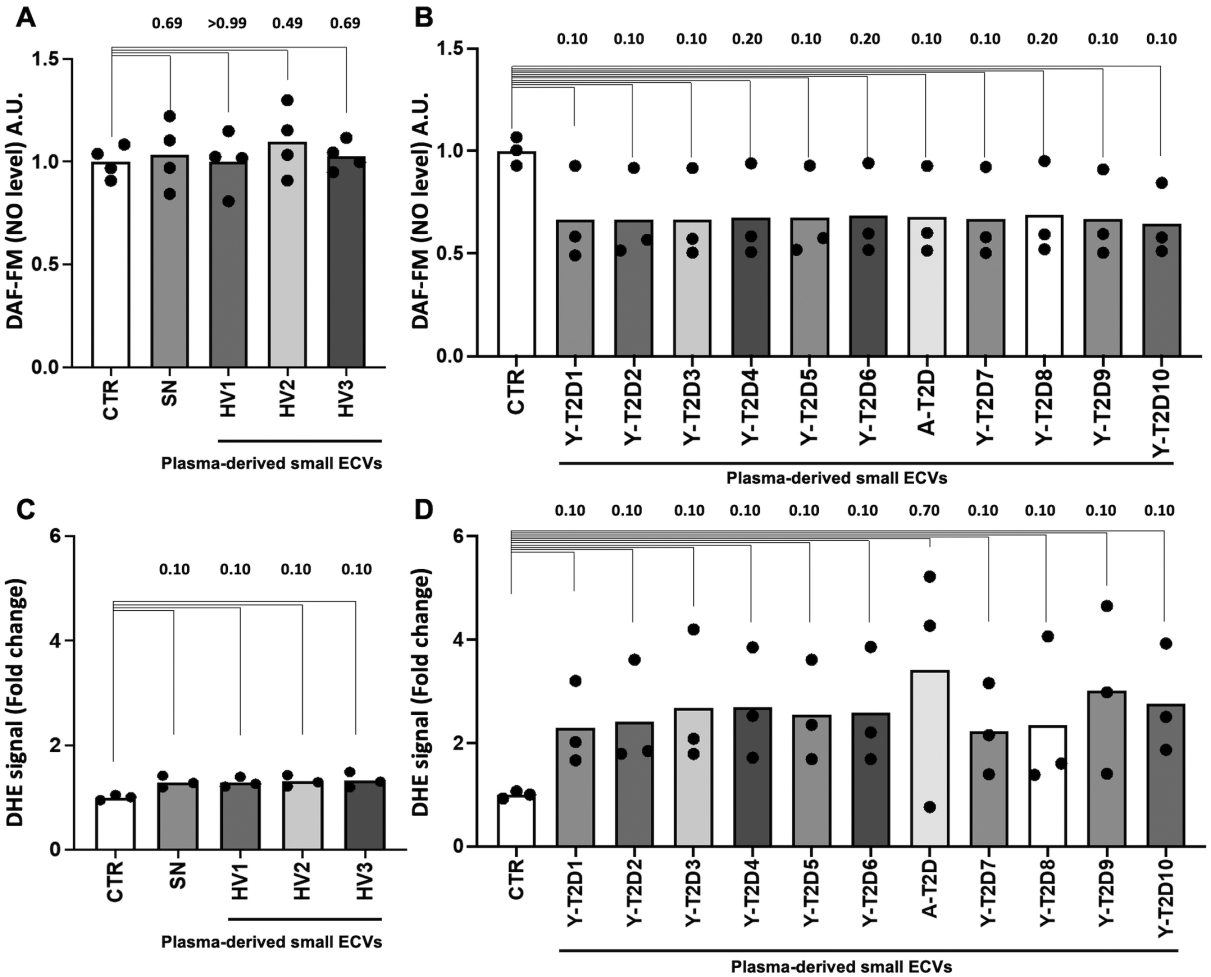
**Analysis of reactive oxygen species (ROS) and nitric oxide levels in HCAECs after small extracellular vesicle (small ECV) treatment.** Small ECVs were isolated from the plasma of healthy donor volunteers (HVs; n=3), youth-onset type 2 diabetes (Y-T2D; n=10), and adult-onset type 2 diabetes (A-T2D; n=1) and subsequently used to treat HCAECs over a period of 48 hours. **A** and **B**, After treatment, flow cytometric analysis of nitric oxide (NO) levels using DAF-FM (4-Amino-5-Methylamino-2',7'-Difluorofluorescein) techniques after treatment with plasma-derived small ECVs. **C** and **D**, Assessment of ROS production using dihydroethidium (DHE) after small ECV treatment. Individual data (black circles) are presented with bar graphs for mean, with 3 replicates for **A** through **D**. Mann Whitney *U* tests were used to compare vs control (CTR, untreated cells) and supernatant (SN; HCAECs treated with SN-depleted small ECVs). *P*<0.05 was considered significant and shown inline.

Protein expression analysis in HCAECs after small ECV treatment from healthy donor volunteers and Y-T2D is shown in Figure 4. There were no statistically significant differences observed in eNOS phosphorylation (Figure 4A), a marker of endothelial dysfunction, between HCAECs treated with plasma-derived small ECVs from healthy volunteers and untreated HCAECs. HCAECs incubated with small ECVs from 10 patients with Y-T2D and 1 patient with A-T2D (serving as a positive control) showed reduced levels of eNOS protein expression compared with untreated HCAECs (Figure 4B). No statistically significant differences were observed in healthy (Figure 4C), but HCAECs treated with Y-T2D small ECVs demonstrated increased protein expression of ICAM, an endothelial membrane–associated inflammatory biomarker (Figure 4D). Flow cytometry analysis further validated the presence of elevated biomarkers of endothelial dysfunction, with decreased NO levels (Figure 5A and 5B) and increased ROS (Figure 5C and 5D) in HCAEC exposed to Y-T2D–derived small ECVs. These results suggest that Y-T2D plasma–derived small ECVs may specifically inhibit eNOS activity, thereby blocking NO generation in HCAECs. Given that the reduced NO bioactivity is a hallmark of endothelial dysfunction, our findings indicate that Y-T2D plasma–derived small ECVs contribute to endothelial dysfunction by inhibiting eNOS activity and reducing NO levels in Y-T2D.

## DISCUSSION

Young adults with type 2 diabetes who were within 5 years of diagnosis exhibited MRI evidence of early CAD in the absence of traditional CAD risk factors such as severe hyperglycemia, marked hypertension, or hyperlipidemia. This evidence was further supported by ex vivo signs of endothelial dysfunction in HCAECs. This clinical translational pilot study provides novel insights into coronary arterial endothelial dysfunction in Y-T2D. Notably, compared with age-matched peers, individuals with Y-T2D experienced vasoconstriction of the right coronary artery and impaired vasodilation of the right brachial artery in response to an isometric handgrip exercise, a reaction typically mediated by NO. Such paradoxical coronary artery vasoconstriction has been observed in adults with established coronary artery disease^23^ but is now described here in youths who were early in their disease course and who did not have marked hyperglycemia or frank elevations in cholesterol concentrations. This clinical response serves as a direct in situ marker of both peripheral and coronary endothelial health in young adults with Y-T2D. Furthermore, we corroborated these imaging findings with functional studies using coronary endothelial cell culture models, marking the first such demonstration in youth. These studies showed induced signaling markers of endothelial dysfunction following incubation with plasma-derived small ECVs from people with Y-T2D.

This study sheds new light on understanding the natural history of Y-T2D and elucidates the earliest pathological features of CAD in a well-characterized cohort of young adults with early metabolic disease. These early coronary proatherogenic changes were detectable and measurable by coronary MRI in this young population. For comparison, CAD in adults with longstanding diabetes had increased CWT on MRI of ≥2.5 mm, and the evidence of coronary endothelial dysfunction required direct invasive coronary catheterization.^9,30,31^ In Y-T2D, 2 studies have shown increased cardiovascular mortality beginning >15 years after diabetes onset.^2,32^ However, few studies have characterized the early stages of CAD, and none have directly examined coronary endothelial function. To date, evidence of impaired vascular aging in Y-T2D has been derived from analysis of large peripheral arteries; Y-T2D has modest increases in aortic arterial stiffness and reduced peripheral endothelial function (BraFMD) compared with age- and BMI-matched peers.^12,13,33–35^ This study extends on and corroborates existing literature by simultaneously evaluating both peripheral and coronary endothelial functions using noninvasive imaging technology in Y-T2D. Our findings reveal dynamic changes in coronary endothelial function, specifically coronary vasoconstriction in the presence of reduced BraFMD, suggesting an increased susceptibility of the coronary endothelium to NO-mediated stimuli.

In our youth cohort, higher HbA1c and BMI were associated with reduced coronary and peripheral vasodilatory response, while HbA1c was also related to CWT. Hyperglycemia alone could induce oxidative stress and contribute to endothelial dysfunction.^10,11^ However, a small pilot study of 7 children with type 1 diabetes demonstrated no significant coronary vasodilation compared with age-matched peers.^36^ In this study, individuals with Y-T2D exhibited a more advanced stage of coronary endothelial dysfunction, as demonstrated by coronary vasoconstriction rather than a mere absence of significant dilation. This indicates that hyperglycemia, along with other factors, may accelerate the early onset of CAD in Y-T2D. These findings underscore the need for further research to elucidate the independent contributions of HbA1c and BMI to endothelial dysfunction in patients with Y-T2D.

These imaging findings were corroborated by ex vivo models that exhibited a phenotype indicative of impaired NO signaling. Our studies demonstrated that Y-T2D small ECVs induced markers of oxidative stress, elevated the proinflammatory endothelial marker ICAM-1, and decreased phosphorylated eNOS activity in cultured HCAECs. Notably, phosphorylated eNOS protein expression levels were significantly lower in treated cells, corresponding to reduced NO production compared with untreated controls. These ex vivo results, together with the imaging evidence of endothelial cell dysfunction, contribute to the growing body of research linking small ECV–mediated ICAM activation to endothelial cell dysfunction and an elevated risk of CAD.^37–39^ This study enhances our understanding of the role of small ECVs in Y-T2D by providing concrete evidence of the functional impact of Y-T2D-derived small ECVs on HCAECs.

### Limitations

Some limitations of this study are noteworthy. The small sample size restricts generalizability; however, this observational study was adequately powered to detect differences between lean healthy individuals and those with Y-T2D using highly reproducible and precise cardiac MRI techniques. Despite the small sample size, these deep phenotyping studies are crucial for understanding the physiological underpinnings of Y-T2D and provide foundational data for designing larger outcome studies of CAD in youth. Furthermore, this translational proof-of-concept study demonstrated the technical feasibility of directly quantifying endothelial structure and function with clinical MRI coupled with ex vivo validation using plasma-derived small ECVs. The small ECV findings supported the phenotype of endothelial dysfunction in Y-T2D although they were limited in scope, as small ECVs from age-matched lean and BMI-matched youth were not available. Including small ECVs from 1 A-T2D served as a positive control for this pilot analysis, but more extensive comparisons with BMI-matched A-T2D participants are necessary. Specifically, we were unable to determine the independent contributions of hyperglycemia, insulin resistance, and obesity to endothelial dysfunction, and our findings cannot be extrapolated to Y-T2D with more severe, longstanding disease. Moreover, observed increases in CWT and coronary vasoconstriction cannot be directly linked to future CAD outcomes.

For the translational study, we utilized a commercial kit for small ECV isolation, which may coisolate nonexosomal entities such as ribonucleoproteins, exomeres, and supermeres. Consequently, the biological effects observed could be attributed to a mixture of these particles and not solely to small ECVs. This highlights the need for cautious interpretation of our findings and suggests the utility of orthogonal isolation methods, such as size-exclusion chromatography or density gradient ultracentrifugation, in future studies to attribute observed effects more precisely to specific extracellular vesicle subtypes. However, the study findings remain valid as our main aims were to demonstrate, noninvasively, early coronary endothelial dysfunction directly on MRI and corroborate biologically in this young population. Further investigations into the biological mechanisms mediating the changes in the HCAEC cells are crucial but were beyond the scope of this analysis. Our study marks an important initial step in elucidating the role of small ECVs in Y-T2D by demonstrating their functional impact on HCAECs. Future studies should compare the effects of small ECVs from Y-T2D and A-T2D to age- and BMI-matched controls exploring potential mediators and mechanisms, including assessments of micro-RNA profiling, proteomics, and lipidomics.

## CONCLUSIONS

This study identified early subclinical coronary atherosclerosis in young adults with Y-T2D, characterized by increased CWT and endothelial dysfunction using noninvasive MRI techniques. Notably, coronary vasoconstriction in the presence of reduced BraFMD suggested an increased susceptibility of the coronary endothelium to NO-mediated stimuli. Additionally, the observed coronary and peripheral arterial pathology was supported by ex vivo experiments with Y-T2D plasma–derived small ECVs, which induced markers of endothelial dysfunction in healthy HCAECs. The integration of MRI assessments of coronary arterial endothelial dysfunction and confirmation and small ECV analysis confirmed the early development of CAD in this young demographic. As the fields of cardiac imaging and small ECVs research continue to advance, a greater emphasis on understanding their crucial roles in Y-T2D, early detection of endothelial dysfunction, and the development of therapeutic interventions is essential.

## ARTICLE INFORMATION

### Acknowledgments

The authors would like to thank their patients, families, and staff at the National Institutes of Health Clinical Center. They also thank Dr Malak Abbas and Amadou Gaye for their expertise in exosomal and statistical analysis, respectively.

### Sources of Funding

S.T. Chung, A.M. Gharib, J. Edwan, K. Z. Abd-Elmoniem, M. Stagliano, and L. Mabundo were supported by the Intramural Program of the National Institute of Diabetes and Digestive and Kidney Diseases. C. Hadigan was supported by the National Institutes of Health Clinical Center.

### Disclosures

None.

### Supplemental Material

Supplemental Methods

Tables S1–S3

Figures S1 and S2

Major Resources Tables

Original uncropped blots

## Supplementary Material


