# Supplementary material for: Endothelial Dysfunction in Youth-Onset Type 2 Diabetes: A Clinical Translational Study
Source: Circ Res. 2024 Jul 29;135(6):639–50. doi: 10.1161/CIRCRESAHA.124.324272 (PMC11361354; doi:10.1161/CIRCRESAHA.124.324272)
Supplement: Supplementary file 1 [file res-135-639-s001.pdf]

Supplemental Methods for small extracellular vesicle Analysis

Characterization of Small Extracellular Vesicles by Transmission Electron Microscopy (TEM):

A 400 mesh Formvar®/carbon-coated copper grid was soaked in 100% ethanol for 1 minute. A 10 µL droplet of small extracellular vesicle samples (in phosphate buffered saline [PBS]) was placed on a sheet of Parafilm, and the grid was floated with the dark-coated side facing the sample, incubating for 90 minutes. The residual sample was washed from the grid by dipping it into 20 µL drops of molecular biology-grade water three times. The grids were then stained for 1 minute with 2% uranyl acetate and allowed to dry for 60 minutes before imaging with a JEM-1200 microscope.

Immunogold Labeling and Transmission Electron Microscopy (TEM) of Small Extracellular Vesicles:

Approximately 10 x 10^9 YD-Ex (as quantified by NTA) were incubated with anti-CD9 and anti-CD63 antibodies for 1 hour at room temperature. A nonspecific IgG isotype antibody served as a negative control. Samples were diluted 1:10 in PBS and incubated with 10.4-nm protein A gold-conjugated secondary anti-rabbit antibody (Nanoprobes, NY, USA) for one hour, followed by crosslinking with 1% glutaraldehyde for 15 minutes, both at room temperature. Sample volumes were brought to 500 µL then filtered through Amicon Ultra 0.5 mL centrifugal filters, MWCO 100 kDa (Millipore Sigma, MO, USA) for buffer exchange according to the manufacturer's recommendations. TEM grids (ultra-thin carbon film supported by Lacey carbon grid 300 mesh, Ted Pella, CA, USA) were glow-discharged (PELCO easiglow, Ted Pella),

applying a current of 35 mA for 60 seconds. After immunogold labeling and chemical cross-linking, 3 µL of purified small extracellular vesicle samples were applied to the glow-discharged TEM grid. The sample was incubated on the grid for 60 seconds for absorption onto the carbon, and excess liquid was blotted using Whatman filter paper No. 1, followed by staining with a 1% phosphotungstic acid solution for 60 seconds. The negatively stained sample was mounted on a room-temperature side-entry holder and loaded into a Tecnai F20 TEM (FEI, OR, USA) equipped with a K2 detector (Gatan, CA, USA). The microscope was operated in low gain mode, and images were acquired with a nominal magnification of 25,000 (corresponding to a pixel size of 1.52 Å / pixel) for a total of 8 seconds (200 ms per frame) with a total dose of 150 electrons / Å^2. Beam-induced motion and stage drift were corrected by whole frame alignments using the MotionCor software tool, bandpass filtered (10 and 200 Å) for visualization purposes and finally analyzed in ImageJ 1.49v (Bethesda, NIH, USA).

Immunoblotting Analysis of Small extracellular vesicle-Specific Markers:

To investigate the presence of small extracellular vesicle protein markers, immunoblotting analysis was performed using intact small extracellular vesicle samples. Determination of small extracellular vesicle concentrations was by a Micro-BCA assay kit (Pierce, Rockford, IL, USA) in accordance with the manufacturer's instructions. The total protein content was calculated by multiplying the protein concentration by the volume of the isolated small extracellular vesicles. Intact small extracellular vesicle samples isolated using the kit method were directly mixed with an equal volume of 2× Laemmli sample buffer. From each prepared sample, 16 µg of protein lysates were separated by 4-12% SDS-PAGE and blotted onto Immobilon-P polyvinylidene difluoride membranes (Millipore, Bedford, MA, USA). Membranes were blocked with 5% skim

milk in PBS containing 0.05% Tween-20 and probed with primary antibodies overnight at 4°C. The antibodies used for immunoblotting were small extracellular vesicle-specific anti-TSG101, anti-CD9, and anti-CD63; Anti-ApoE was used to confirm the absence of lipoproteins potentially included in the extraction process; and anti-calnexin, a non-small extracellular vesicle endoplasmic reticulum-related antibody, was used as per Minimum information for studies of extracellular vesicles 2018 guidelines[25]. After the membranes were washed three times with PBS containing 0.05% Tween-20 (PBST) for 10 minutes, they were incubated for 1 hour with goat anti-rabbit IgG and HRP-conjugated antibody (#7074; 1:3000; Cell Signaling Technology, Danvers, MA, USA). All membranes were washed three times for 10 minutes with PBST. The signals of the HRP-conjugated antibodies were developed using Western blotting detection reagent (Western Femto ECL Kit, Invitrogen, USA) and visualized using a chemiluminescence detection system (ChemiDoc Bio-Rad, Hercules, CA, USA). To further verify the purity of small extracellular vesicle isolation, immunoblotting analysis was performed on small extracellular vesicles from healthy donors and Y-T2D cell lysate from HCAEC was used as a negative control for purity analysis.

Human Coronary Artery Endothelial Cell Cultures

Primary HCAECs were cultured for a maximum of four passages in endothelial basal medium-2 (EBM-2) supplemented with growth factors [endothelial growth medium EGM and TM-2 MV Microvascular Endothelial Cell Growth Medium-2 BulletKit from Lonza (Walkersville, MD, USA) containing 2% serum]. Cells were maintained at 37°C in a humidified incubator with 5% CO2 and 95% air. Primary HCAECs were seeded at a density of 70,000 cells/well in 12-well plates or 150,000 cells/well in 6-well plates for protein analysis, respectively. HCAEC donors (n

= 4) ranged in age from 19 to 32 years old and cells were purchased from Lonza and Lifeline (Frederick, MD, USA). Mycoplasma contamination testing was performed by Lonza and Lifeline. HCAECs were then cultured with the endothelial basal medium prepared in small extracellular vesicles depleted fetal bovine serum (FBS) purchased from (Gibco, Waltham, MA, USA).

Tracing Small extracellular vesicle Uptake in the Ex Vivo System of HCAECs

Small extracellular vesicle staining was conducted using SYTO-RNASelect dye (SYTO® RNASelect™ stain for RNA staining or BODIPY® TR ceramide for membrane staining, SYTO® RNASelect™ Green Fluorescent Cell Stain (S32703). HCAECs cultured in chamber slides (Nunc™ Lab-Tek™ II Chamber Slide™ System), then 100 µL labeled small extracellular vesicles were diluted in 400 µL EBM2 endothelium growth medium and incubated with HCAECs for 24h and 48h. The HCAECs were then labeled with Phalloidin (CST, 8953) (1:200) at room temperature for 15 minutes, followed by staining with Prolong® Gold Antifade Reagent with DAPI. The cells were then visualized using a fluorescent microscope.

HCAEC Incubation and Western Blot Analysis of Nitric Oxide and Inflammatory Markers

Whole cell protein lysates were isolated from HCAECs treated with plasma-derived small extracellular vesicles from 11 plasma samples at a final concentration of 50 µg/mL or from PBS-control untreated cells for 48 hours using RIPA buffer (Thermo Scientific Pierce, Rockford, IL, USA) supplemented with protease and phosphatase inhibitor cocktail (Thermo Scientific Pierce). Protein lysates (30 µg) were resolved by SDS-PAGE and transferred to a nitrocellulose membrane with Trans-Blot (Invitrogen). Membranes were incubated with 5% blocking solution (Bio-Rad; nonfat milk) in PBS-Tween 20 (0.1%) for 1 hour at room temperature and then

incubated overnight at 4°C with primary antibodies. For Western blots, loading control-normalized densitometry results were analyzed, but FCs from the control condition are plotted for ease of interpretation. Antibodies against peNOS (no. 9571, 1:1000), eNOS (no. 9572, 1:1000) were used.

HCAEC Incubation and Measurement of Reactive Oxygen Species

HCAECs were seeded into 6-well plates and exposed to small extracellular vesicles from participants for 4 hours. Dihydroethidium (2.5 µM), a redox-sensitive fluorescent dye, was applied to cells for 30 minutes at 37°C. Cells were washed three times with warm PBS, EBM2 growth media was added, and cells were collected by scraping before analysis by flow cytometer (Fortessa, NHLBI, NIH, Bethesda, USA). Light scatter parameters were set to eliminate dead cells and subcellular debris. The red ethidium signal was measured, and oxidative stress was estimated using the mean fluorescence intensity (MFI) of the population. Auto-fluorescence gains were determined in unlabeled cells and set at the first logarithmic decade.

HCAEC Incubation and Measurement of Nitric Oxide

HCAECs were seeded into 6-well plates and exposed to small extracellular vesicles from participants for 48 hours. A NO-specific probe (DAF-FM DA) was applied to cells for 20–60 minutes at temperatures ranging from 4℃ to 37℃. HCAECs were not trypsinized. Cells were washed 3 times with warm PBS1X to remove excess probe. Fresh medium was then added to the HCAECs, and they were incubated for an additional 30 minutes to allow complete desiccation of the intracellular diacetates. MFI was measured at fluorescence excitation and emission of 488 and 530/30 nm respectively by flow cytometry.

Probing of Plasma-Derived Small extracellular vesicles Transfer to Target HCAECs

PKH26 lipid dye was diluted in 100 µL diluent C to a final concentration of 8 µM (dye solution). Then 10 µg of small extracellular vesiclesin 20 µL DPBS were diluted with 80 µL diluent C, added to the dye solution, and incubated for 5 minutes while mixed with gentle pipetting. Excess dye was bound with 100 µL 10% small extracellular vesicle-depleted fetal bovine serum (Sigma-Aldrich) in Dulbecco's modified Eagle's medium (Sigma-Aldrich) as previously described [28]. Small extracellular vesicles were washed twice by centrifugation (13,000 g, 60 min). Interaction of PKH26-labeled small extracellular vesicles and target ECs was assessed by flow cytometry (Fortessa, Becton Dickinson, USA).

Nanoparticle Tracking Analysis

Nanoparticle tracking analysis (NTA) from Malvern (NanoSight NS300, Malvern Instruments, Malvern, USA) was used for size distribution and concentration measurements of small extracellular vesicle samples in liquid suspension from the properties of both light scattering and Brownian motion. The NanoSight NS300 with a 405-nm laser instrument (Malvern Instruments, Malvern, USA) was used to detect nanovesicles. Five videos of typically 60-second duration were taken. Data were analyzed using the FlowJo software. The Brownian motion of each particle was tracked using the Stokes–Einstein equation: $D = kT/6\pi\eta r$, where D is the diffusion coefficient, $kT/6\pi\eta r = f0$ is the frictional coefficient of the particle, for the special case of a spherical particle of radius r moving with uniform velocity in a continuous fluid of viscosity η, k is Boltzmann's constant, and T is the absolute temperature.

Major Resources Table

Antibodies

Target antigen	Vendor or Source	Catalog #	Working concentration	Lot # (preferred but not required)	Persistent ID / URL
**Western Blot**					
β-Actin	Sigma	A3854	1:50000		AB_262011
CD63	Cell Signaling Technology	#52090	1:1000		AB_2924771
CD9	Cell Signaling Technology	#13174	1:1000		AB_2798139
TSG101	Cell Signaling Technology	#72312	1:1000		AB_2927716
CD81	Cell Signaling Technology	#56039	1:1000		AB_2924772
Alix	Cell Signaling Technology	#2171	1:1000		AB_2299455
Anti-Calnexin	Cell Signaling Technology	2433	1:1000		AB_2243887
ApoE Antibody	Cell Signaling Technology	#68587	1:1000		AB_3094528
eNOS Antibody	Cell Signaling Technology	#9572	1:1000		AB_329863
Phospho-eNOS	Cell Signaling Technology	#9571	1:1000		AB_329837
Anti-ICAM1	Cell Signaling Technology	#4915	1:1000		AB_2280018
Dihydroethidium	Thermo Fisher Scientific	D11347	2.5 µM		NA
DAF-FM Diacetate	Thermo Fisher Scientific	D23844	5 µM		NA
SYTO ® RNASelectTM Green	Thermo Fisher Scientific	S32703	10 µM		NA

Fluorescent Cell Stain					
Alexa Fluor® 594 phalloidin	Thermo Fisher Scientific	A12381	0.165 µM		NA
ProLong™ Gold Antifade Mountant with DNA Stain DAPI	Thermo Fisher Scientific	P36935	One drop		NA
PKH26 Red Fluorescent Cell	Sigma	MIDI26	8 µM		NA
CD63	Invitrogen	10628D	1:1500		AB_2532983
CD9	Santa Cruz Biotechnology,	sc-13118,	1:500		AB_627213

Cultured Cells

Name	Vendor or Source	Sex (F, M, or unknown)	Persistent ID / URL
Human Coronary Artery Endothelial Cells	LONZA, CC-2585	F	44948
Human Coronary Artery Endothelial Cells	LONZA, CC-2585	F	41911
Human Coronary Artery Endothelial Cells	LONZA, CC-2585	M	40418
Human Coronary Artery Endothelial Cells	LONZA, CC-2585	M	50684

Other

Description	Source / Repository	Persistent ID / URL
Human Plasma from Whole Blood	LONZA, 3W-811	N/A

NO animals or DNA/ DNA clones were used in this study.

Supplemental Figure 1. Participant Flow Diagram

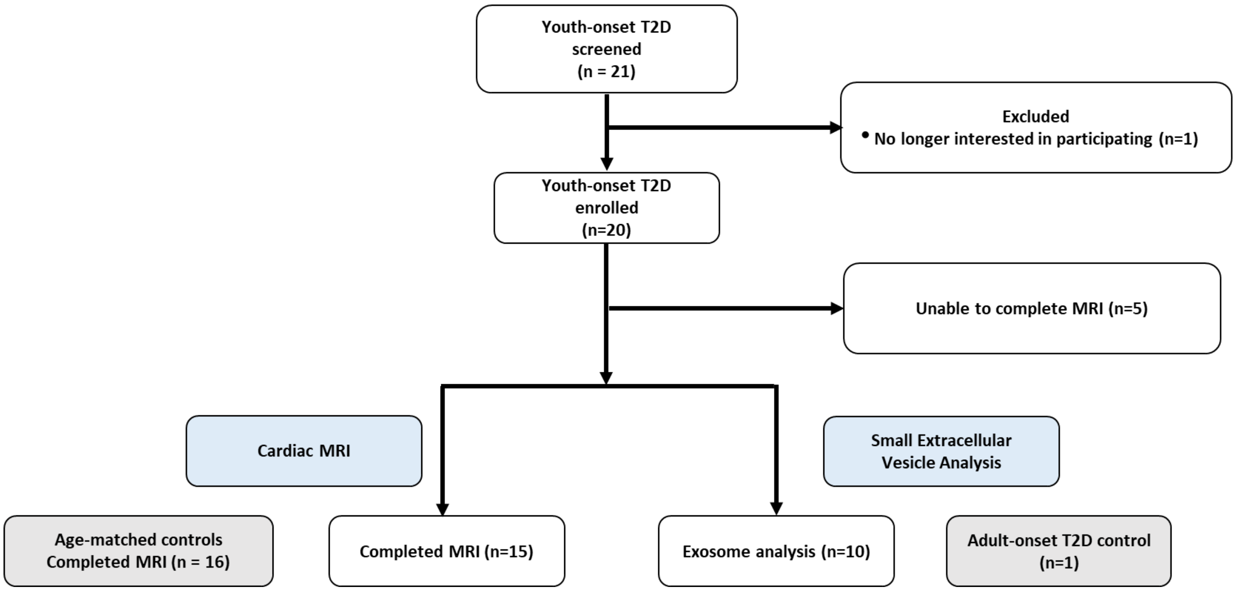

Supplemental Figure 2

Characterization of plasma-derived small extracellular vesicles from healthy volunteers and Y-T2D. Plasma-derived small extracellular vesicles were isolated from healthy lean and T2D volunteers. Western blot analysis was conducted on lysates sourced from the small extracellular vesicle fraction and cell pellet, utilizing antibodies against CD81 and Alix.

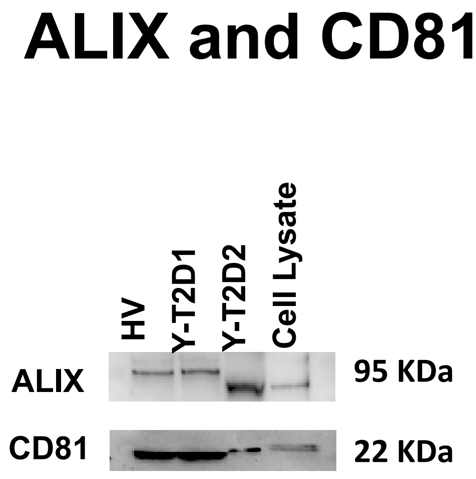

Supplementary Table 1. Demographic and cardiometabolic characteristics in lean and youth-onset type 2 diabetes with and without imaging.

	Controls (n=16)	Y-T2D (n=20)	Y-T2D MRI (n=15)
**Age (years)**	22.8±2.6	19.4±1.3	19.5±1.4
**Female**	8 (50)	10 (50)	8 (53)
**BMI (kg/m2)**	23.4±3.4	37.8±8.4	35.9±7.4
**African American race**	10 (63)	16 (72)	13 (87)
**Prediabetes diagnosis**	0 (0)	2 (20)	1 (7)
**Duration of diabetes (years)**	-	2.7±2.0	2.8±2.0
**History of smoking**	2 (15)	0 (0)	0 (0)
**Systolic BP (mmHg)**	114±11	128±14	128±13
**Diastolic BP (mmHg)**	65±8	72±10	72±8
**Heart Rate (beats/min)**	73±9	82±14	83±14
**Metabolic characteristics**			
**Hemoglobin A1c (%)**	5.0±0.5	6.4±1.1	6.1±0.8
**hs-C-Reactive Protein (mg/L)**	0.5 (0.2, 1.8)	2.7 (1.4, 9)	2.3 (1, 7.2)
**Total cholesterol (mg/dL)**	162±25	151±25	146±24
**LDL cholesterol (mg/dL)**	82±21	88±19	86±19
**Medication Use**			
**Anti-hypertensive agents**	0(0)	5 (20)	4 (28)
**Metformin**	0 (0)	11 (55)	9 (60)
**GLP-1 agonist**	0 (0)	12 (60)	11 (73)
**Insulin**	0(0)	5 (25)	3 (20)
**Statin or lipid lowering**	0(0)	0(0)	0(0)

Y-T2D: youth-onset type 2 diabetes entire cohort, Y-T2D MRI: youth-onset type 2 diabetes with cardiac imaging, hsCRP: high sensitivity C-Reactive protein. LDL: low density lipoprotein, GLP-1: glucagon like-1 peptide, Data are mean±SD, median (25th-75th percentile) or n (%).

Supplementary Table 2. Demographic and cardiometabolic characteristics in volunteers who had small extracellular vesicle analysis.

	Y-T2D (n=10)	A-T2D (n=1)
**Age (years)**	19.1±1.0	40
**Female**	5 (50)	1 (100)
**BMI (kg/m2)**	34.3±6.86	45.5
**African American race**	9 (90)	1 (100)
**History of smoking**	0 (0)	0(0)
**Systolic BP (mmHg)**	126.8±15.52	180
**Diastolic BP (mmHg)**	71.9±7.87	104
**Heart Rate (beats/min)**	77.2±16.51	84
**Prediabetes diagnosis**	2 (20)	0 (0)
**Hemoglobin A1c (%)**	5.8±0.5	7.7
**Hemoglobin A1c (mmol/mol)**	40±5.5	61
**Fasting glucose (mg/dL)**	100.8±21.7	147
**Fasting insulin (mcU/mL)***	31.3 (4.5, 111.5)	28.1
**HsCRP (mg/L)**	4.7 (0.7, 19.4)	9.4
**Total cholesterol (mg/dL)**	151.6±23.87	187
**LDL cholesterol (mg/dL)**	88.3±17.59	114
**HDL cholesterol (mg/dl)**	42±8.47	36
**Triglyceride (mg/dL)**	76.4 (45, 126)	218
**High LDL≥130 mg/dL**	0 (0)	0 (0)

**Anti-hypertensive agents**	4(40)	1 (100)
**Metformin use**	7 (70%)	1 (100)
**GLP-1 agonist use**	6(60)	0 (0)
**Insulin use**	1(10)	0 (0)
**Vessel wall thickness (mm)**	1.31±0.13	Unable to complete
**Coronary artery flow mediated dilation (%)**	-1.06±17.1	Unable to complete
**Brachial artery flow mediated dilation (%)**	3.4±17.2	Unable to complete

Y-T2D: youth-onset type 2 diabetes, hsCRP: high sensitivity C-Reactive protein. LDL: low density lipoprotein, HDL: high density lipoprotein, GLP-1: glucagon like-1 peptide. Data are mean±SD, median (25-75th percentile), or n (%). The A-T2D participant attempted the cardiac magnetic resonance imaging but was unable to complete the study because of claustrophobia.

Supplemental Table 3. Relationship of coronary vessel wall thickness, endothelial function, and cardiometabolic markers

	**Right coronary wall thickness n=31 r (*P*-value)**	**Right coronary artery dilation n=31 r (*P*-value)**	**Right brachial artery dilation n=31 r (*P*-value)**
**Hemoglobin A1c (%)**	**0.37 (0.04)**	**-0.47 (0.01)**	**-0.50 (0.01)**
**Body mass index (kg/m^2)**	0.19 (0.3)	-0.30 (0.11)	**-0.37 (0.04)**
**Systolic blood pressure* (mmHg)**	0.10 (0.60)	-0.29 (0.13)	-0.35 (0.07)
**Diastolic blood pressure* (mmHg)**	0.12 (0.52)	-0.19 (0.32)	-0.36 (0.06)
**Heart rate* (Beats/min)**	0.17 (0. 39)	-0.17 (0.39)	-0.31 (0.12)
**High sensitivity C-Reactive protein (mg/L)**	0.12 (0.52)	-0.19 (0.33)	-0.116 (0.53)
**Fasting glucose (mg/dL)**	-0.04 (0.84)	-0.33 (0.07)	-0.13 (0.47)
**LDL cholesterol (mg/dL)**	-0.15 (0.43)	-0.11 (0.53)	-0.14 (0.44)
**Total cholesterol (mg/dL)**	-0.35 (0.06)	0.27 (0.14)	0.03 (0.89)
**Triglycerides (mg/dL)**	-0.12 (0.52)	-0.22 (0.22)	-0.25 (0.17)

Data are spearman correlation coefficients r (P-value) *n=28 (control, n=13; Y-T2D, n=15)
