# Supplementary material for: Endothelial Dysfunction in Youth-Onset Type 2 Diabetes: A Clinical Translational Study
Source: Circ Res. 2024 Jul 29;135(6):639–50. doi: 10.1161/CIRCRESAHA.124.324272 (PMC11361354; doi:10.1161/CIRCRESAHA.124.324272)
Supplement: Supplementary file 2 [file res-135-639-s002.pdf]

Major Resources Table

In order to allow validation and replication of experiments, all essential research materials listed in the Methods should be included in the Major Resources Table below. Authors are encouraged to use public repositories for protocols, data, code, and other materials and provide persistent identifiers and/or links to repositories when available. Authors may add or delete rows as needed.

Animals (in vivo studies)

Species	Vendor or Source	Background Strain	Sex	Persistent ID / URL

Genetically Modified Animals

	Species	Vendor or Source	Background Strain	Other Information	Persistent ID / URL
**Parent - Male**					
**Parent - Female**					

Antibodies

Target antigen	Vendor or Source	Catalog #	Working concentration	Lot # (preferred but not required)	Persistent ID / URL
**Western Blot**					
β-Actin	Sigma	A3854	1:50000		AB_262011
CD63	Cell Signaling Technology	#52090	1:1000		AB_2924771
CD9	Cell Signaling Technology	#13174	1:1000		AB_2798139
TSG101	Cell Signaling Technology	#72312	1:1000		AB_2927716
CD81	Cell Signaling Technology	#56039	1:1000		AB_2924772
Alix	Cell Signaling Technology	#2171	1:1000		AB_2299455
Anti-Calnexin	Cell Signaling Technology	2433	1:1000		AB_2243887
ApoE Antibody	Cell Signaling Technology	#68587	1:1000		AB_3094528
eNOS Antibody	Cell Signaling Technology	#9572	1:1000		AB_329863
Phospho-eNOS	Cell Signaling Technology	#9571	1:1000		AB_329837
Anti-ICAM1	Cell Signaling Technology	#4915	1:1000		AB_2280018
Dihydroethidium	Thermo Fisher Scientific	D11347	2.5 µM		NA
DAF-FM Diacetate	Thermo Fisher Scientific	D23844	5 µM		NA

SYTO ® RNASelectTM Green Fluorescent Cell Stain	Thermo Fisher Scientific	S32703	10 µM		NA
Alexa Fluor® 594 phalloidin	Thermo Fisher Scientific	A12381	0.165 µM		NA
ProLong™ Gold Antifade Mountant with DNA Stain DAPI	Thermo Fisher Scientific	P36935	One drop		NA
PKH26 Red Fluorescent Cell	Sigma	MIDI26	8 µM		NA
CD63	Invitrogen	10628D	1:1500		AB_2532983
CD9	Santa Cruz Biotechnology,	sc-13118,	1:500		AB_627213

DNA/cDNA Clones

Clone Name	Sequence	Source / Repository	Persistent ID / URL

Cultured Cells

Name	Vendor or Source	Sex (F, M, or unknown)	Persistent ID / URL
Human Coronary Artery Endothelial Cells	LONZA, CC-2585	F	44948
Human Coronary Artery Endothelial Cells	LONZA, CC-2585	F	41911
Human Coronary Artery Endothelial Cells	LONZA, CC-2585	M	40418
Human Coronary Artery Endothelial Cells	LONZA, CC-2585	M	50684

Data & Code Availability

Description	Source / Repository	Persistent ID / URL

Other

Description	Source / Repository	Persistent ID / URL
Human Plasma from Whole Blood	LONZA, 3W-811	N/A

ARRIVE GUIDELINES

The ARRIVE guidelines (https://arriveguidelines.org/) are a checklist of recommendations to improve the reporting of research involving animals. Key elements of the study design should be included below to better enable readers to scrutinize the research adequately, evaluate its methodological rigor, and reproduce the methods or findings.

Study Design

Groups	Sex	Age	Number (prior to experiment)	Number (after termination)	Littermates (Yes/No)	Other description
Group 1 (Control)						
Group 2						
Add more if needed						

Sample Size: Please explain how the sample size was decided Please provide details of any a *prior* sample size calculation, if done.

Inclusion Criteria

Exclusion Criteria

Randomization

Blinding

DOI [to be added]
