# Supplementary material for: Endothelial Dysfunction in Youth-Onset Type 2 Diabetes: A Clinical Translational Study
Source: Circ Res. 2024 Jul 29;135(6):639–50. doi: 10.1161/CIRCRESAHA.124.324272 (PMC11361354; doi:10.1161/CIRCRESAHA.124.324272)
Supplement: Supplementary file 4 [file res-135-639-s004.pdf]

Figure 2J

A

TSG101

Set1

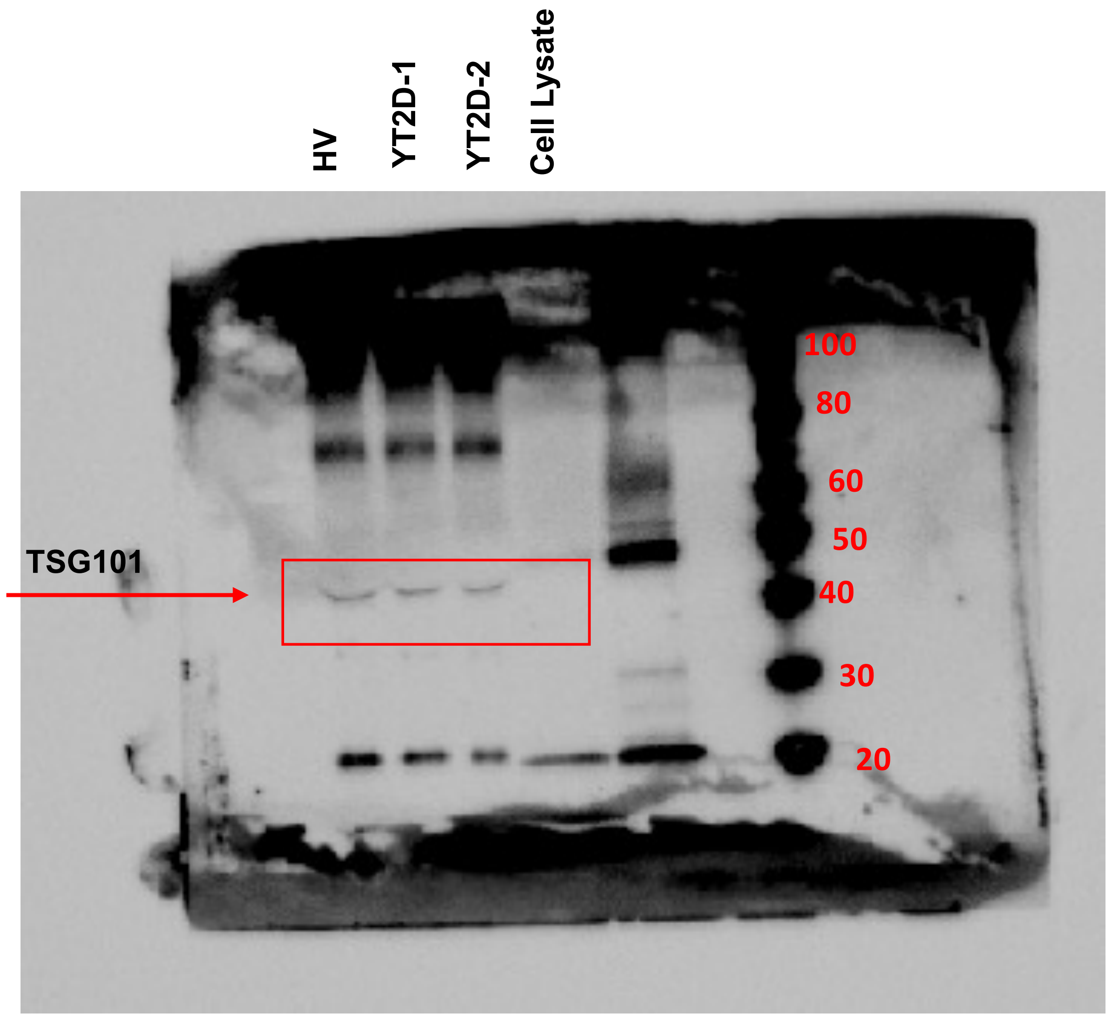

Set2

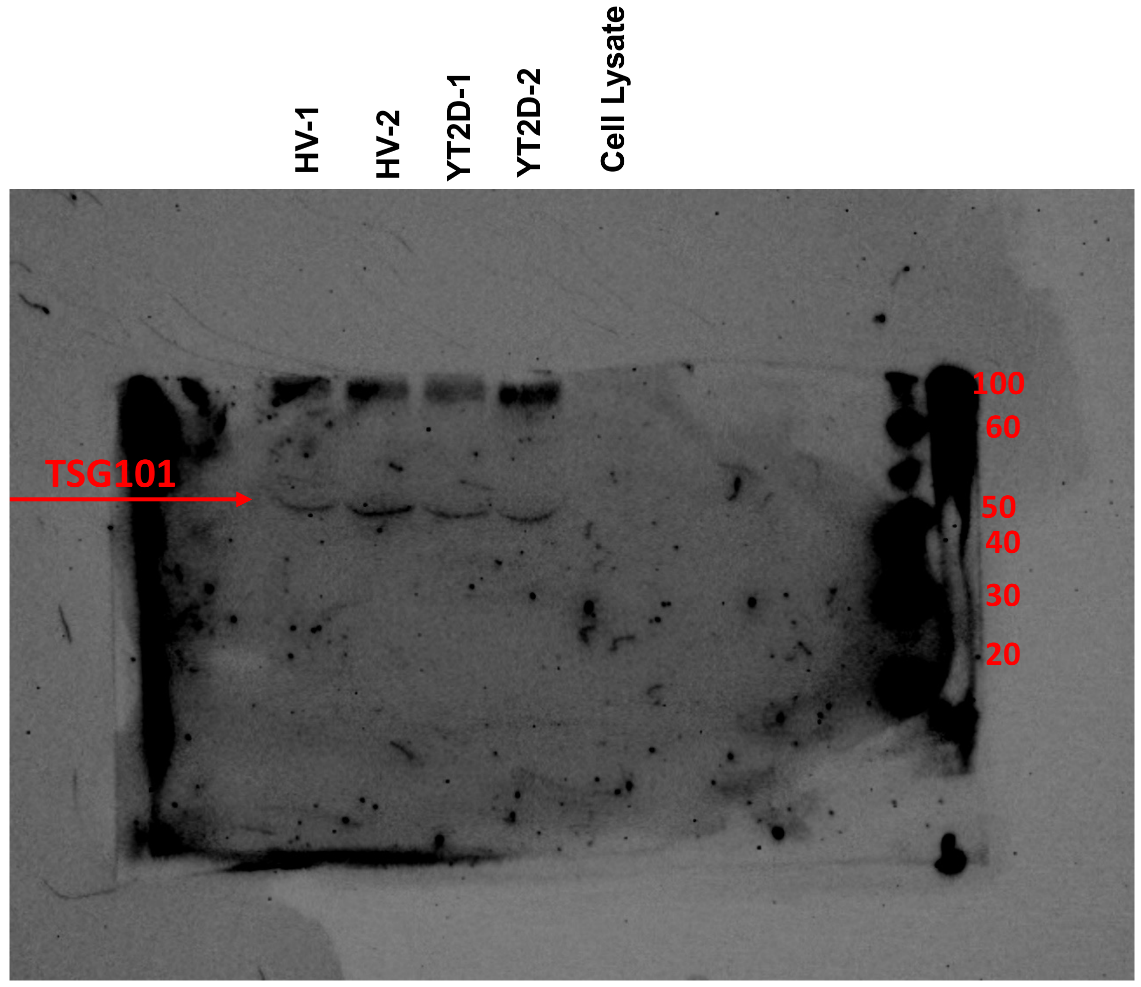

Full uncropped immunoblots of (A) TSG101 and (B) CD9 from Figure 2J. The red rectangle indicates the portion of the blot displayed in the manuscript. N=2

Figure 2J

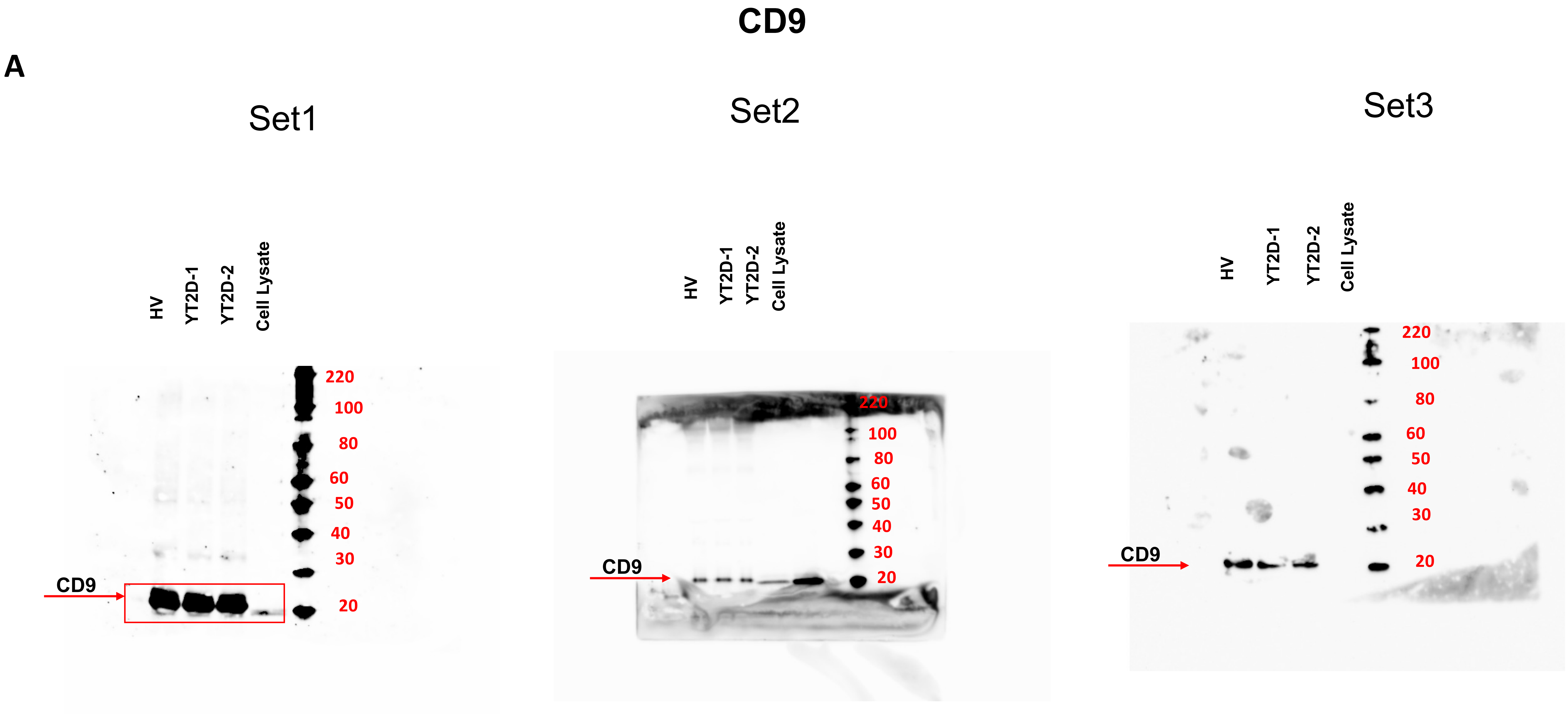

Full uncropped immunoblots of CD9 (A) from Figure 2J. The red rectangle indicates the portion of the blot displayed in the manuscript. N=3

Figure 2J

CD63

A

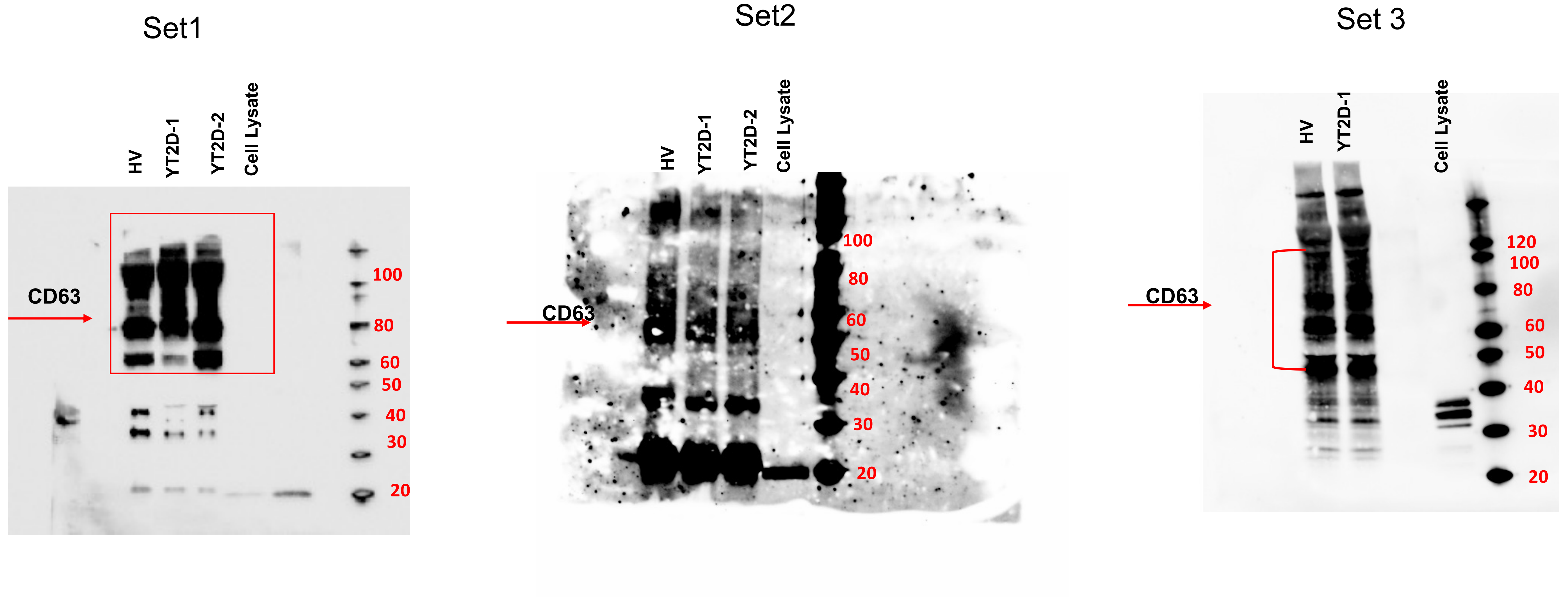

Full uncropped immunoblots of CD63 (A) from Figure 2J. The red rectangle indicates the portion of the blot displayed in the manuscript. N=3

Figure 2J

ApoE

A

Set 1

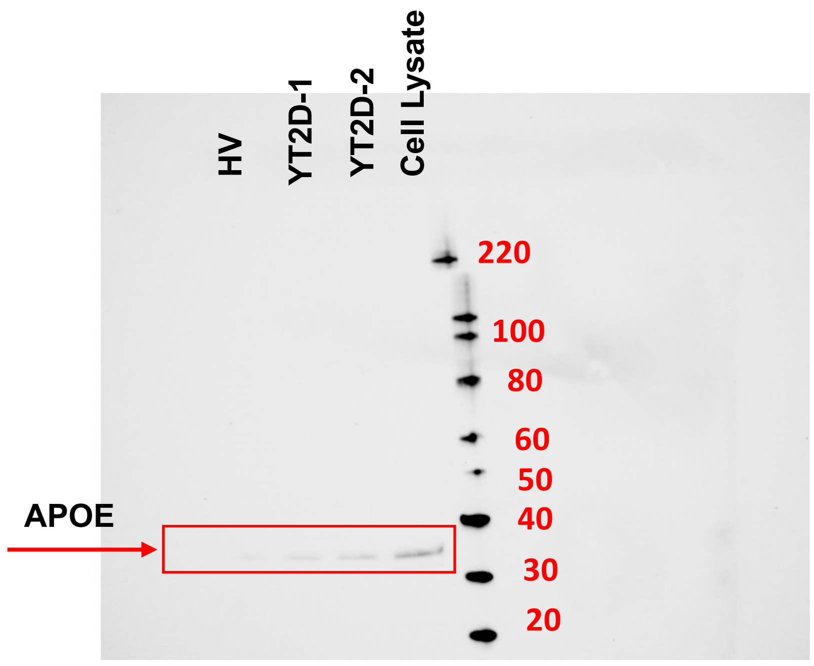

Set 2

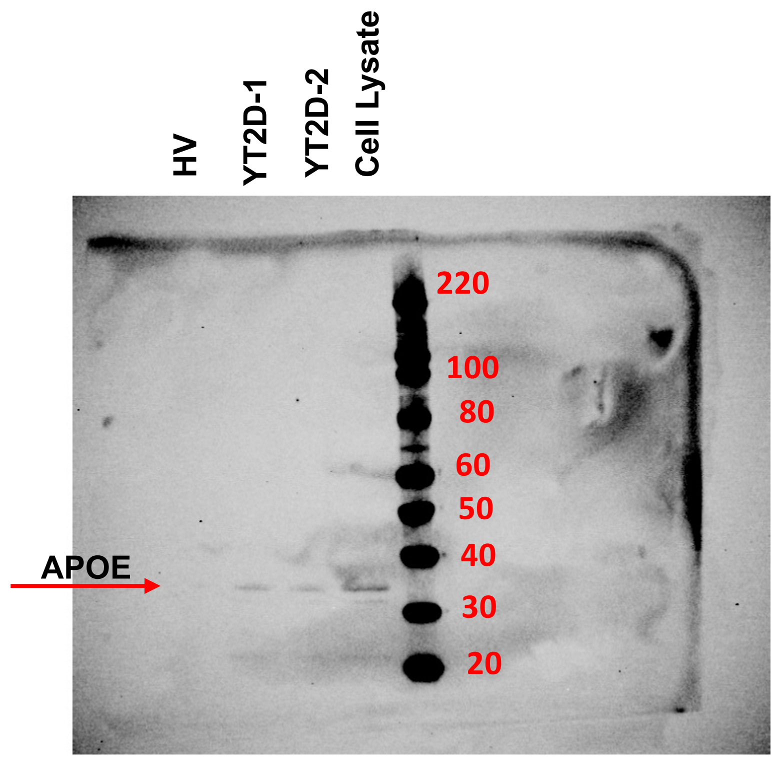

B

ALIX and CD81

Set1

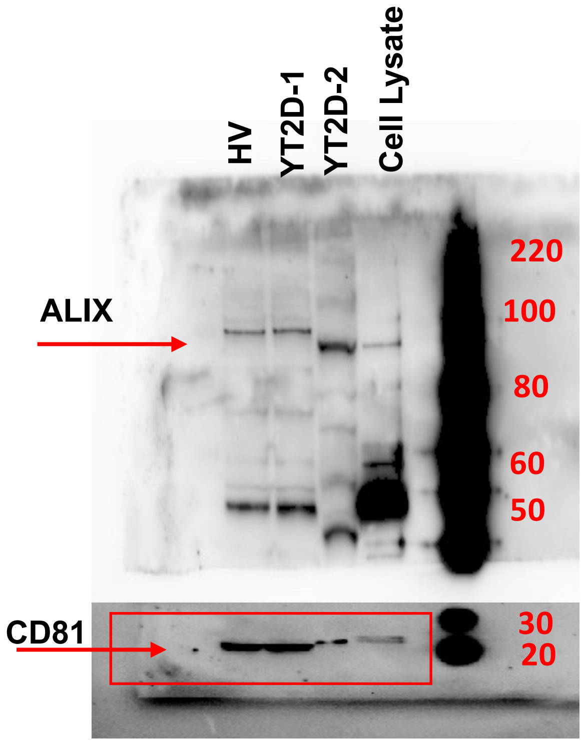

Full uncropped immunoblots of ApoE (A) from Figure 2J. The red rectangle indicates the portion of the blot displayed in the manuscript. N=2

Full uncropped immunoblots of CD81 and ALIX (B). N=1

Figure 2J

A

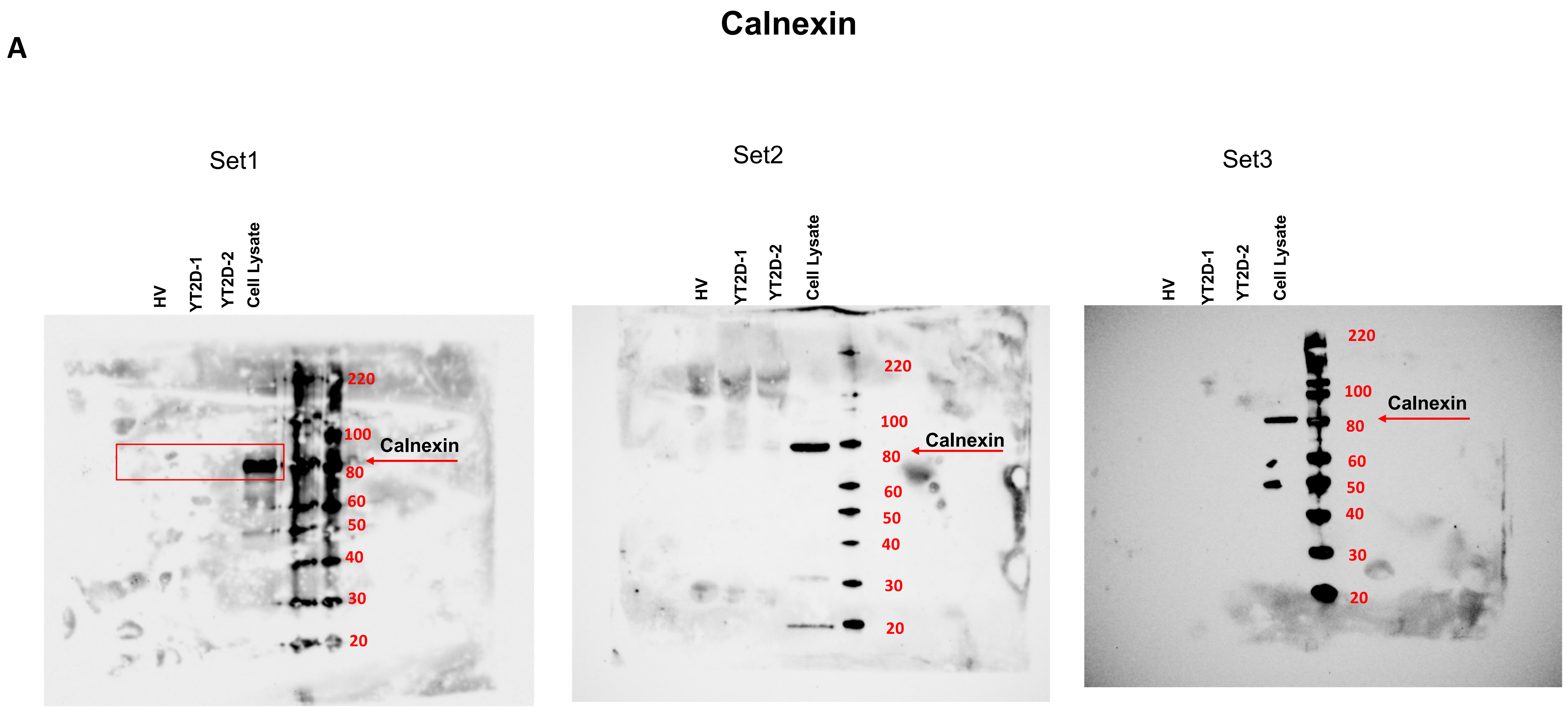

Full uncropped immunoblots of Calnexin (A) from Figure 2J. The red rectangle indicates the portion of the blot displayed in the manuscript. N=3

Figure 4A

peNOS

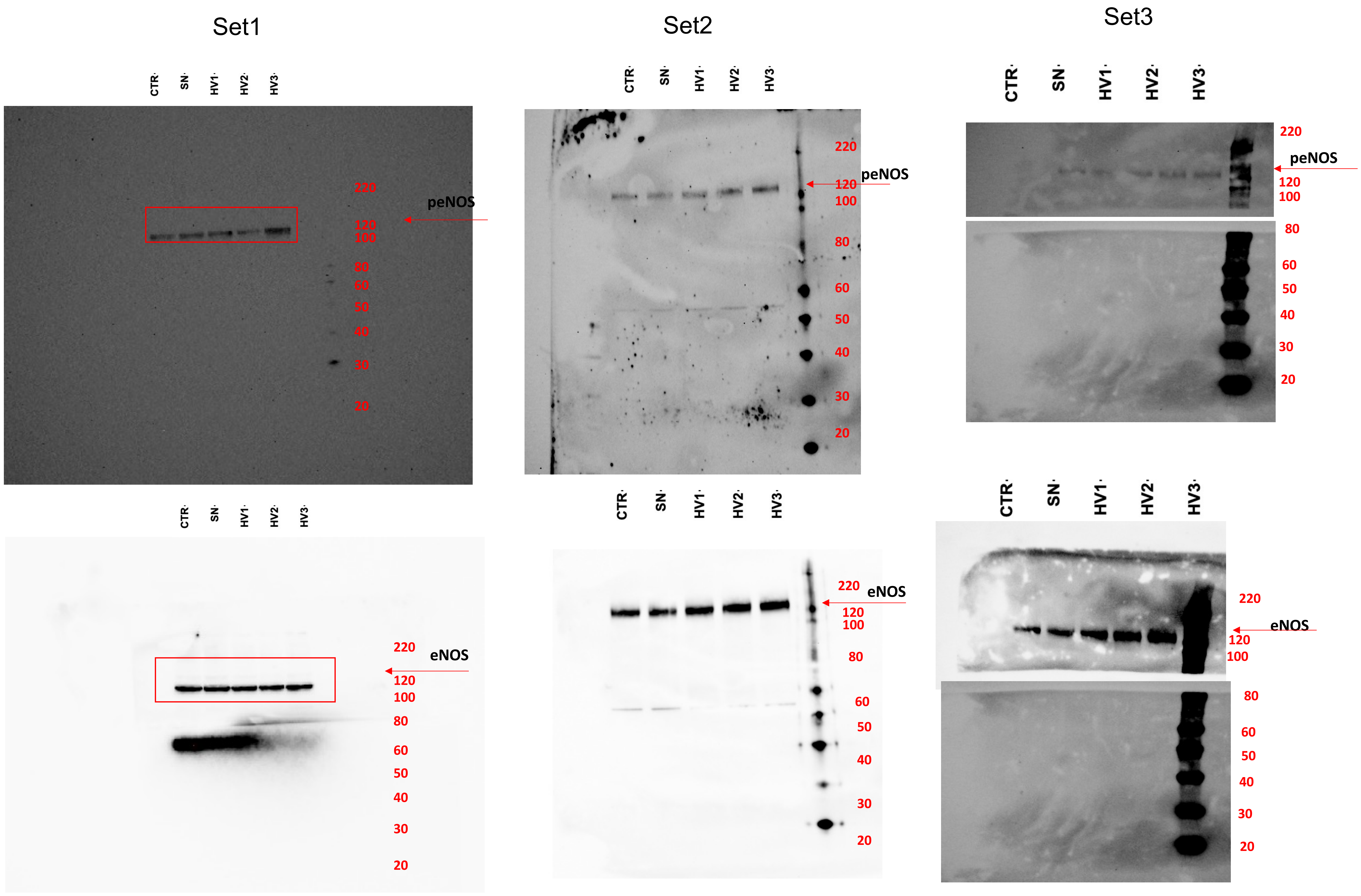

Uncropped full-length pictures of western blotting membranes for peNOS presented in the manuscript Fig4.
The red rectangle indicates the portion of the blot displayed in the manuscript. N=3

Figure 4B

peNOS

Set1 Set2

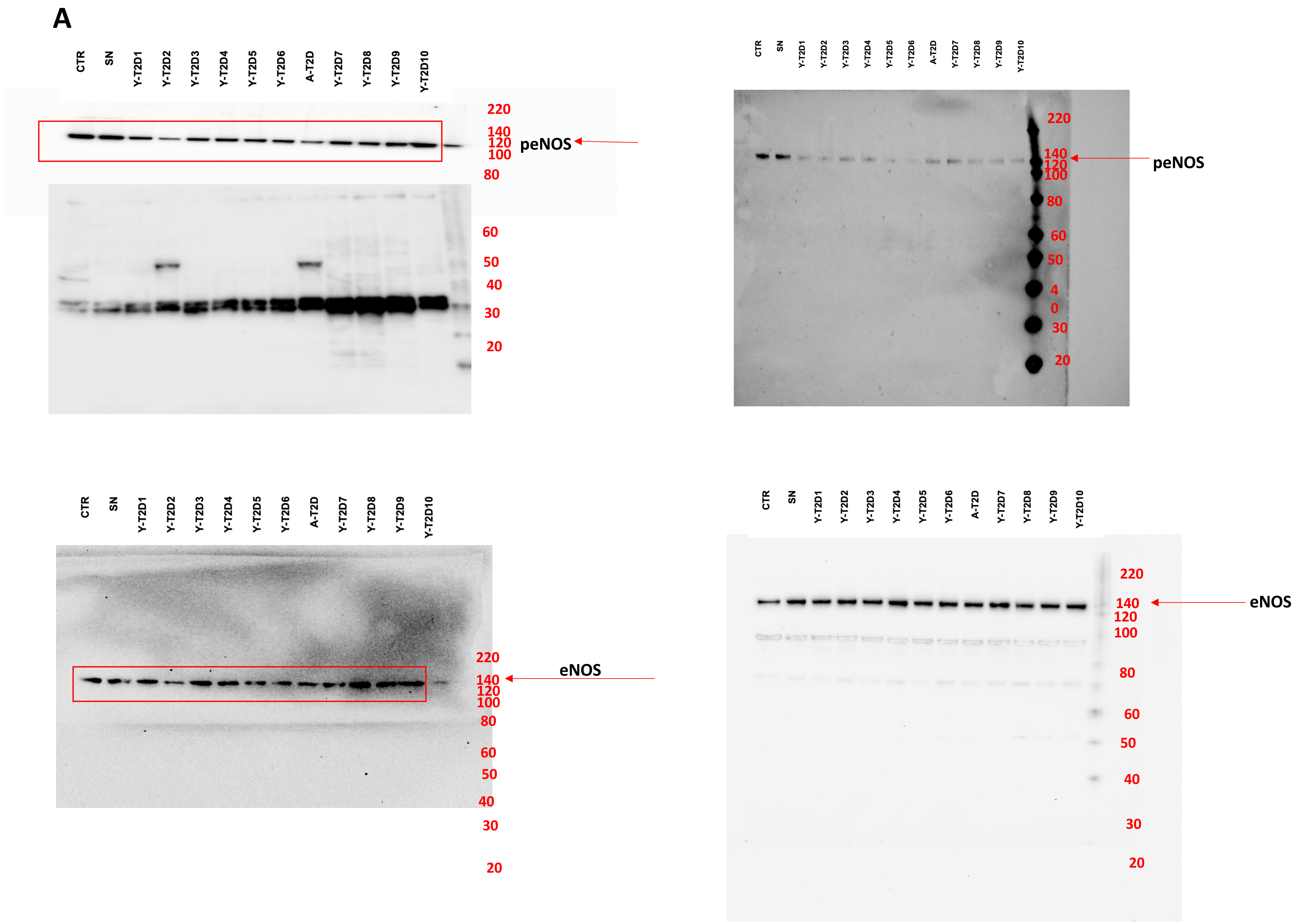

Uncropped full-length pictures of western blotting membranes presented in the manuscript Fig4. The red rectangle indicates the portion of the blot displayed in the manuscript set1 and set2. N=2

Figure 4B

peNOS

Set3

Set4

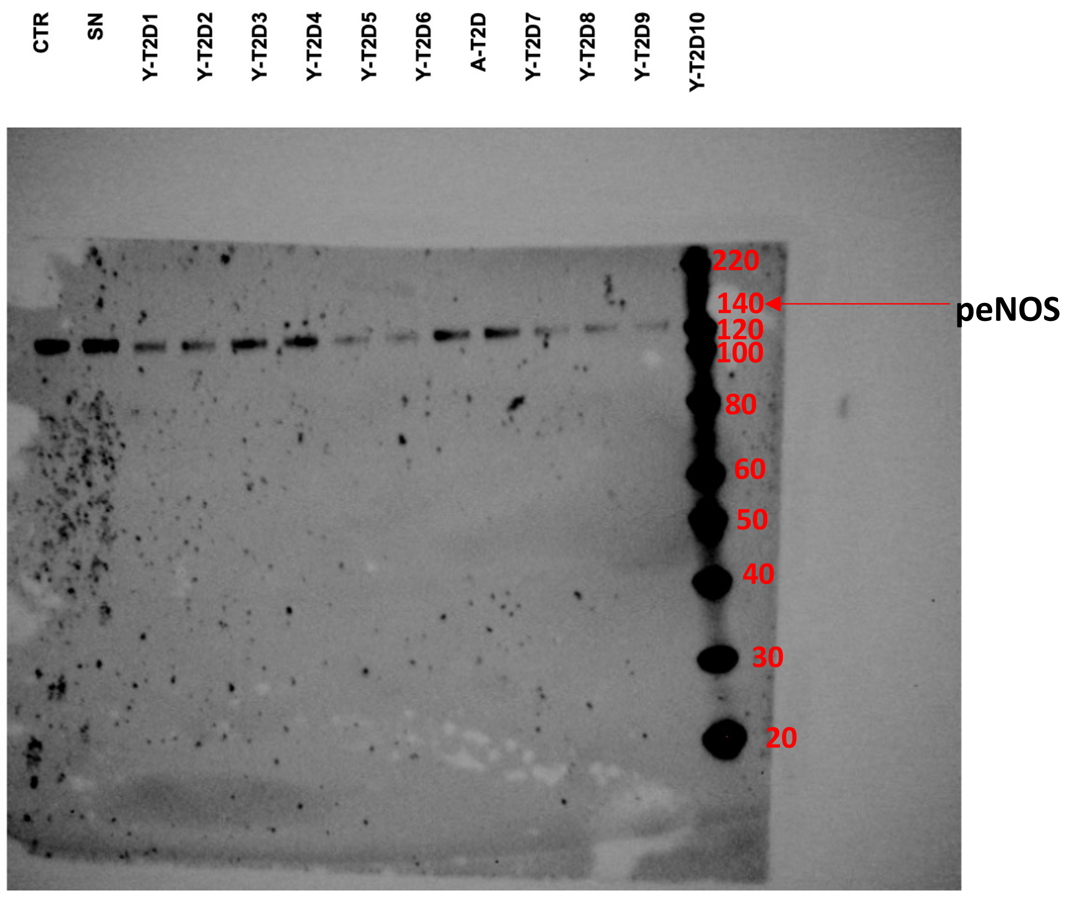

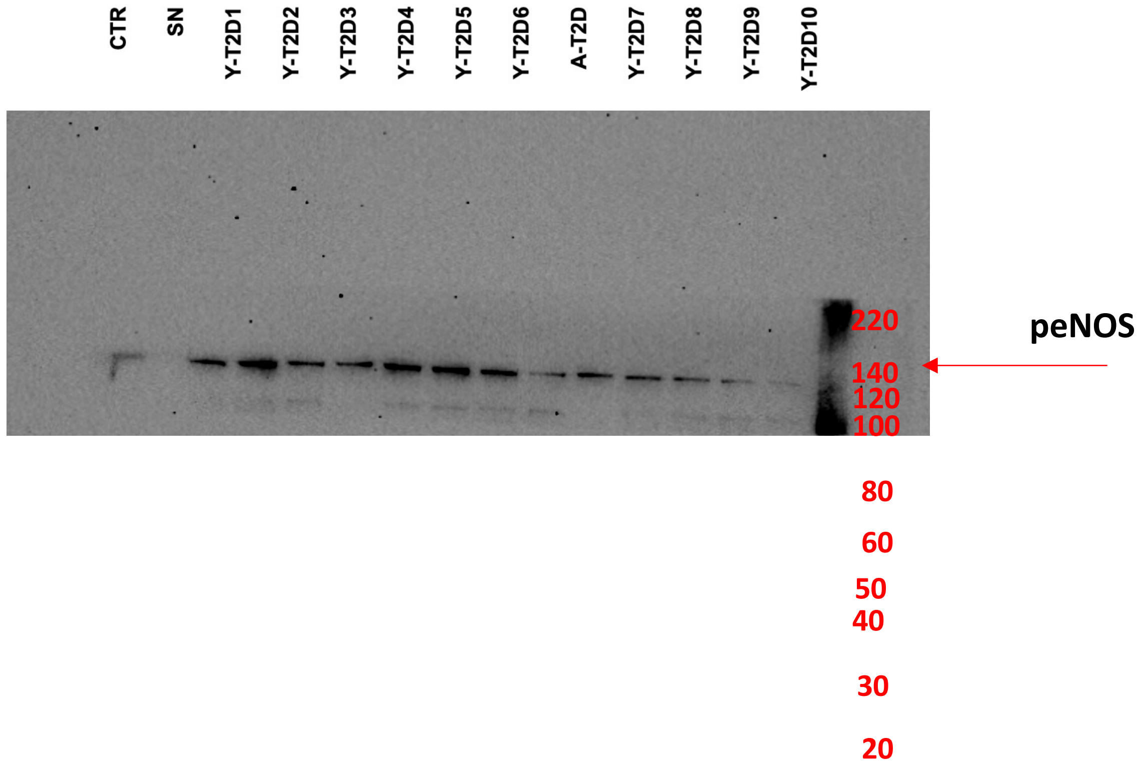

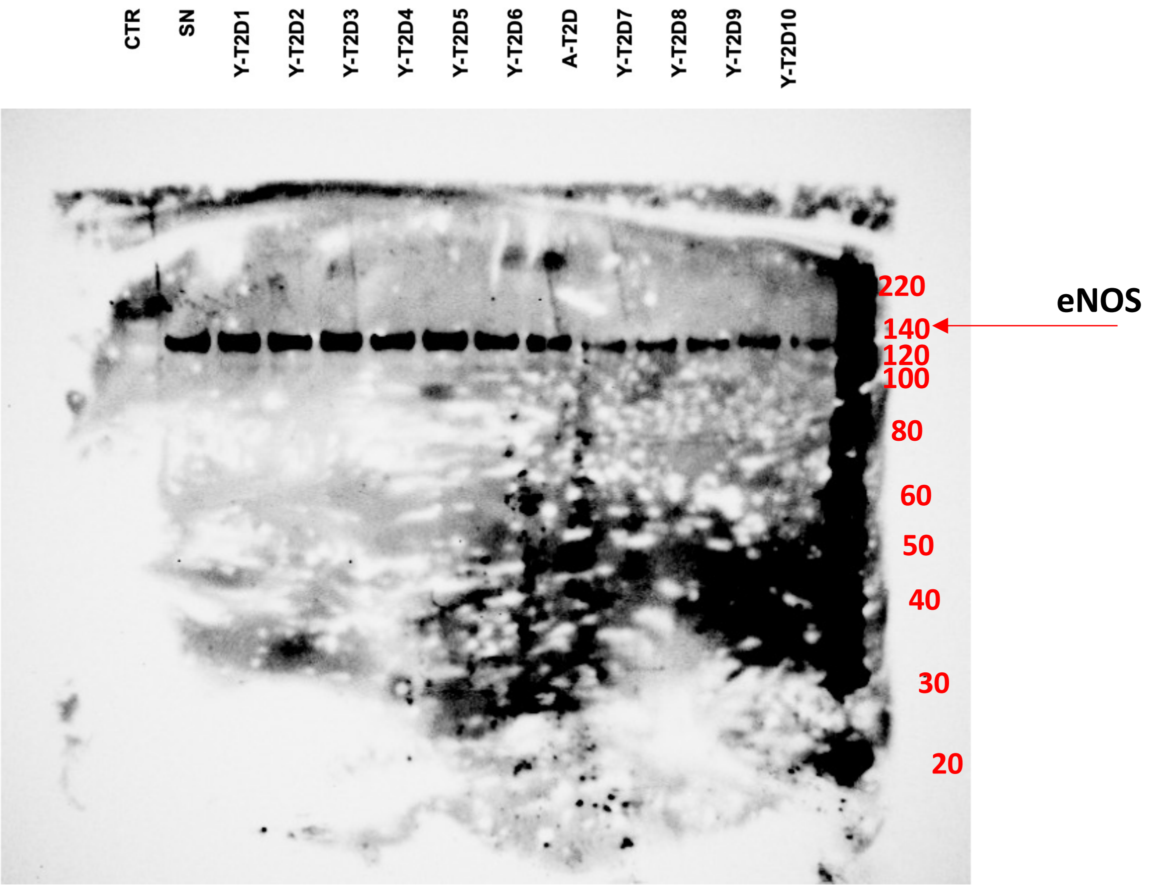

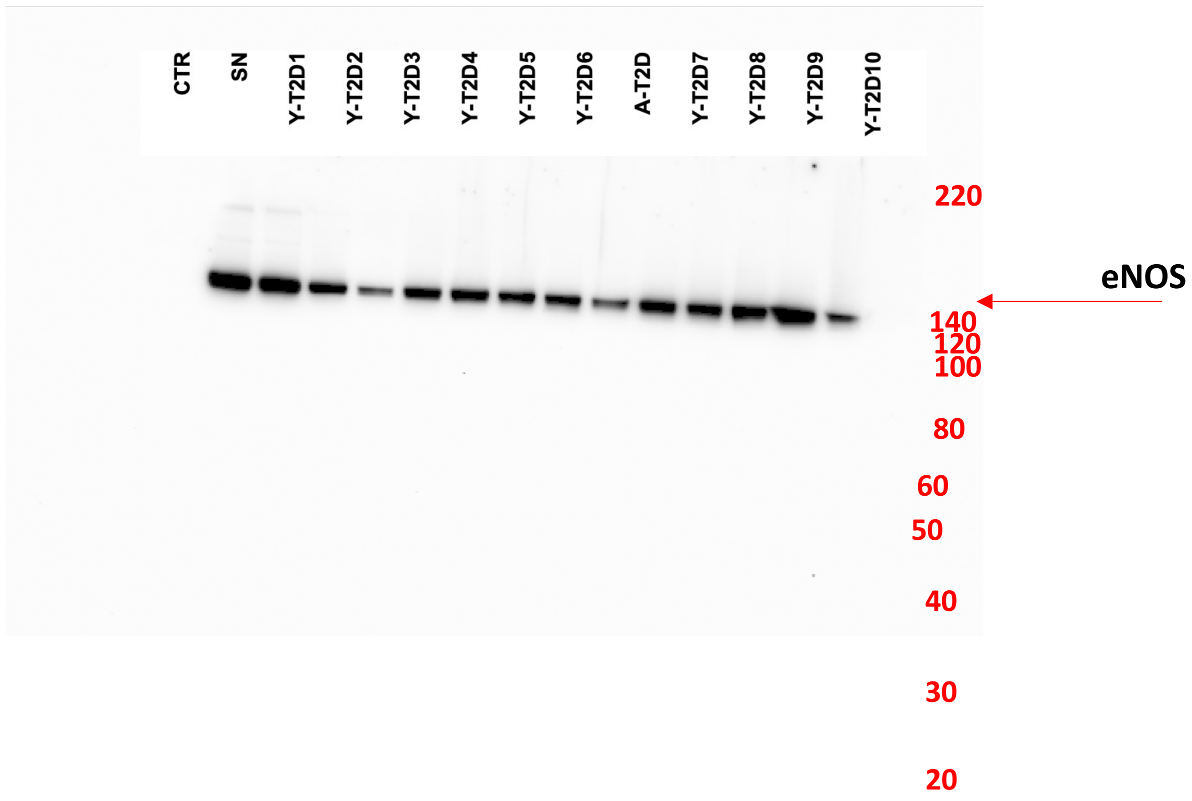

Uncropped full-length pictures of western blotting membranes for peNOS used for quantification for set3 and set4. N=2

Figure 4C

ICAM

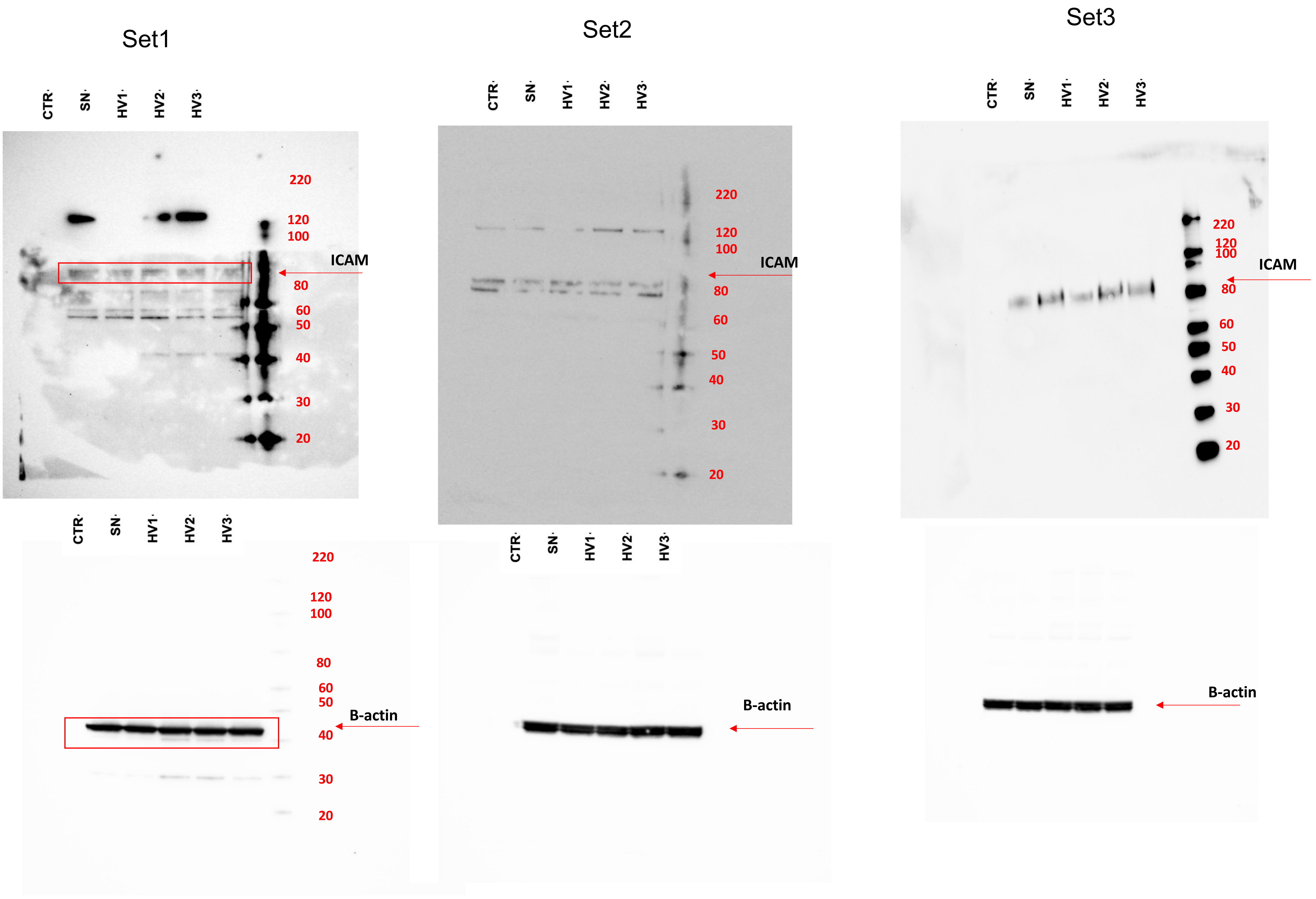

Uncropped full-length pictures of western blotting membranes for ICAM presented in the manuscript Fig4. The red rectangle indicates the portion of the blot displayed in the manuscript. N=3

Figure 4D

ICAM

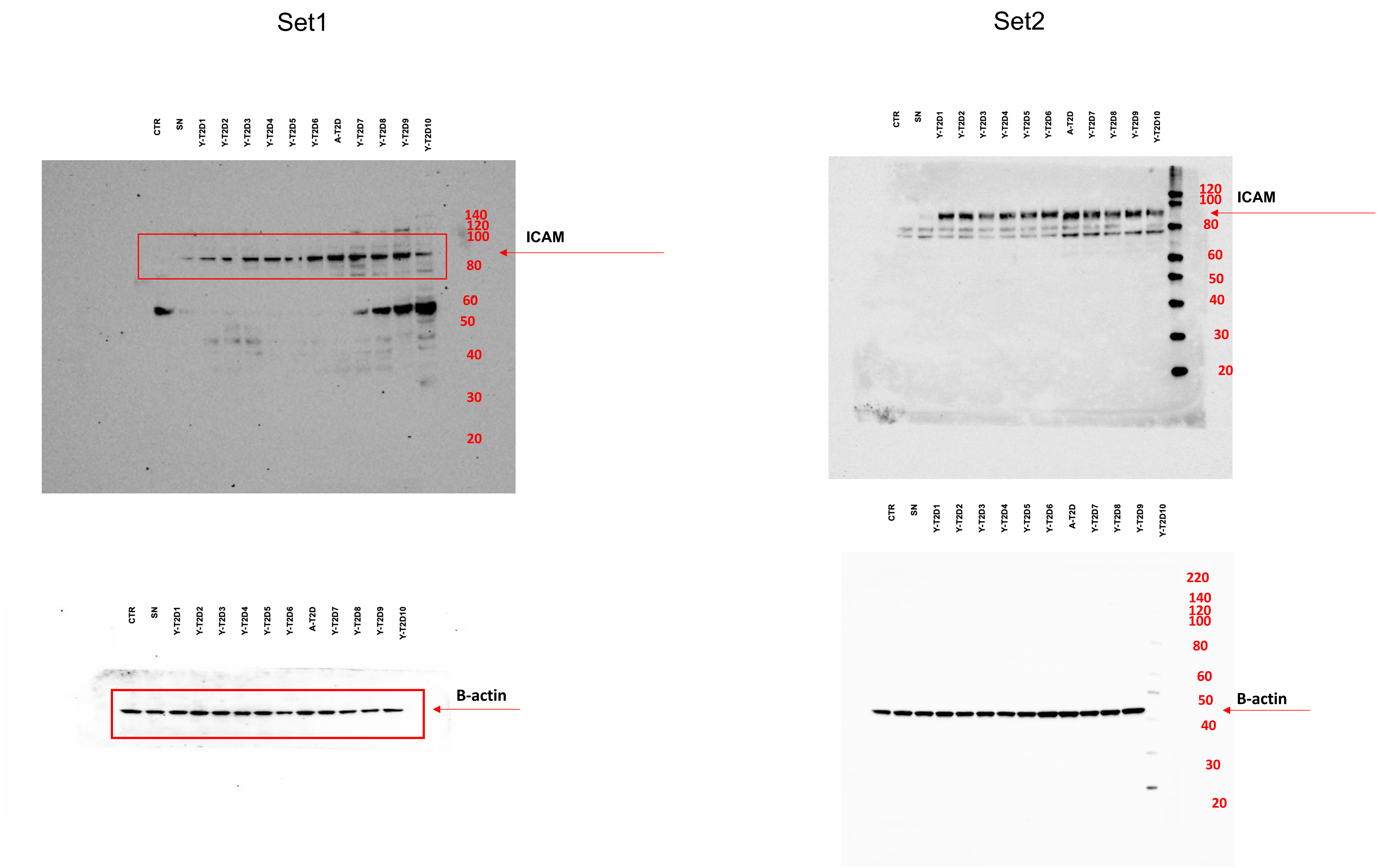

Uncropped full-length pictures of western blotting membranes for ICAM presented in the manuscript Fig4. The red rectangle indicates the portion of the blot displayed in the manuscript. Set1 and Set2. N=2

Figure 4D

ICAM

Set3 | Set4

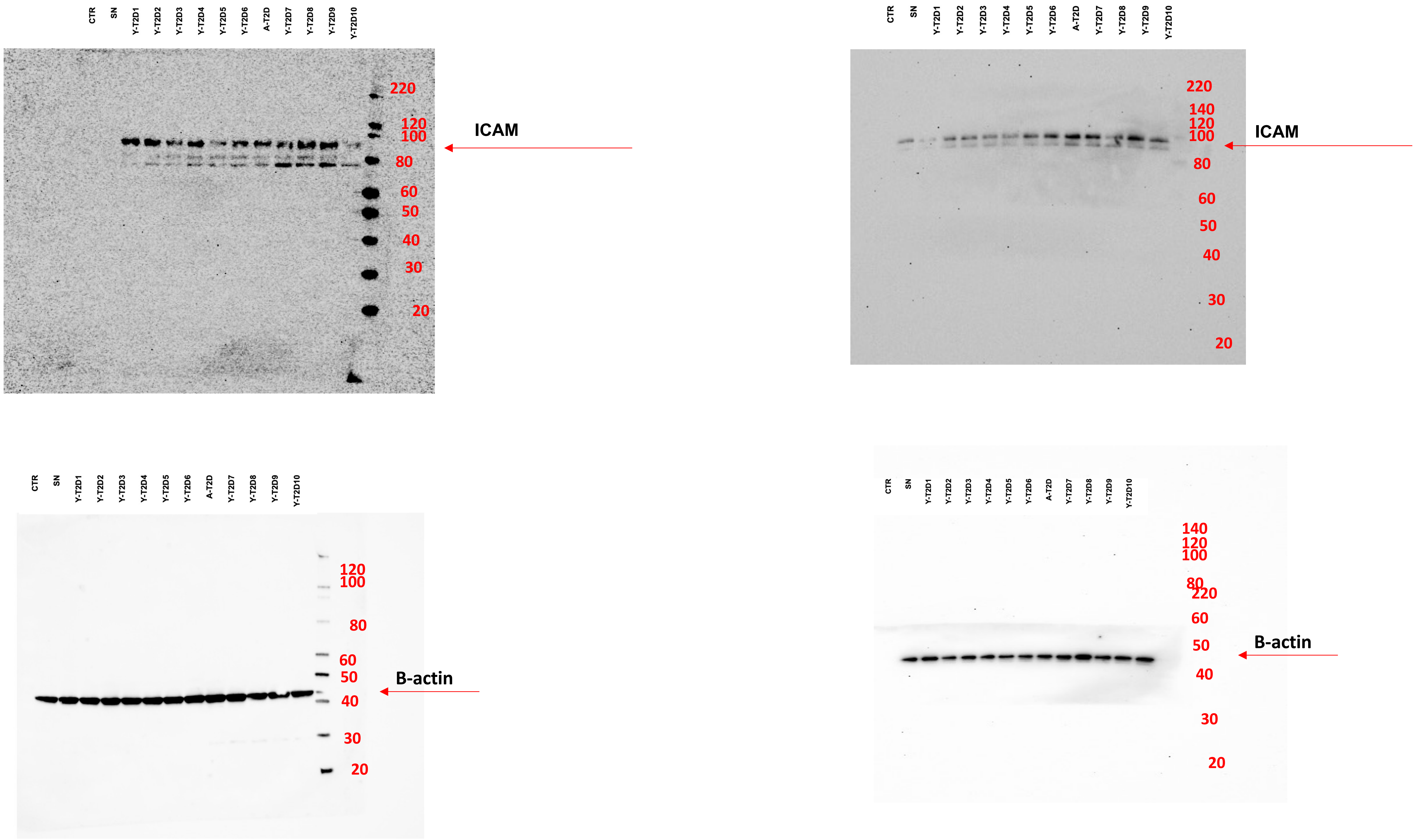

Uncropped full-length pictures of western blotting membranes for ICAM used for quantification for set3 and set4. N=2
